# The sulfur content and isotopic composition of the subarc mantle

**DOI:** 10.1126/sciadv.aeb9747

**Published:** 2026-07-31

**Authors:** Zoltán Taracsák, Tamsin A. Mather, Terry Plank, Ally Peccia, Shuo Ding, Brian Monteleone, Alessandro Aiuppa, David M. Pyle

**Affiliations:** ^1^ Department of Earth Sciences, University of Cambridge, Cambridge CB2 3EQ, UK.; ^2^ Department of Earth Sciences, University of Oxford, Oxford OX1 3AN, UK.; ^3^ Lamont-Doherty Earth Observatory, Columbia University, Palisades, NY 10964-8000, USA.; ^4^ Department of Geological Sciences, University of Florida, Gainesville, FL 32611-2120, USA.; ^5^ Department of Geology and Geophysics, Woods Hole Oceanographic Institute, Woods Hole, MA 02543, USA.; ^6^ Dipartimento di Scienze Della Terra e Del Mare, Università degli Studi di Palermo, 90123 Palermo, Italy.

## Abstract

Sulfur plays a critical role in modulating redox cycling on Earth. Yet, sulfur’s behavior during subduction and in mantle redox reactions is debated. We analyzed ^34^S/^32^S in mafic arc melt inclusions from contrasting subduction zones and modeled slab-mantle interaction to investigate the subduction zone sulfur cycle. We find that degassing may enrich or deplete the melt strongly in ^34^S as a function of melt redox state. After correction for this effect, arc magmas have a substantially narrow range of δ^34^S values (+3 ± 2‰), higher than the ambient upper mantle (−1‰). Slab-derived sulfur is oxidized, evidenced by a concurrent increase in mantle Fe^3+^ and sulfur contents, and contributes up to 86% of the mantle wedge’s sulfur budget. Arc magma δ^34^S values reflect a common slab source for sulfur: the oceanic crust. Subduction zones act as a “filter” for oxidative power and ^34^S, effectively returning these to the surface over geological timescales.

## Teaser

Sulfur isotopes in volcanic glasses reveal the global significance and the unexpectedly uniform source of arc magma sulfur.

## INTRODUCTION

The transfer of volatile elements hosted in subducting slabs, including H, C, S, and Cl, into the mantle wedge under volcanic arcs has a profound influence on Earth’s evolution. Water addition drives melting under arcs (*1*–*3*), while the high water (H_2_O) contents of arc magmas affect melt evolution (*4*) and magma storage (*5*, *6*). Furthermore, the elevated Cl and S contents of arc magmas are key to ore-forming processes (*7*), and their atmospheric injection via volcanic eruptions leads to climatic and environmental impacts (*8*). Subduction zone processes also control the amount of volatiles that reach the deeper mantle, with important implications for mantle melting, heterogeneity, volatile enrichment and fluxes, and magma redox in intraplate settings (*9*–*11*). While the importance of material transfer between subducting slabs and the mantle wedge is widely accepted, the recycling efficiency and the relative importance of individual source lithologies (serpentinites, oceanic crust, and marine and terrigenous sediments) during the geochemical cycling of different volatiles remain less constrained. This includes the subduction zone sulfur (S) cycle.

S is present in surface environments in both sulfide (S^2−^) and sulfate (SO_4_^2−^) minerals (*12*), while in silicate melts, it can be dissolved as S^2−^ and as SO_4_^2−^ anions. In magmas, these two redox states occur within an oxygen fugacity (*f*o_2_) range relevant to modern magmatic systems on Earth (*13*–*15*), making S an important electron acceptor or donor during mantle melting, fractionation, and degassing (*11*, *16*, *17*). The multivalent nature of S makes Earth’s magmas unique in the Solar System compared to other bodies like the Moon, where highly reducing conditions prevent the formation of S^6+^, and Mars, where conditions are comparatively reduced (*18*) and sulfide is stabilized even at more oxidizing conditions by high melt iron(II) oxide (FeO) contents (*19*). S contents in parental arc magmas vary from ∼1000 μg/g, similar to mid-ocean ridge basalts (MORBs), to >7000 μg/g in the Trans-Mexican Volcanic Belt and have been suggested to correlate with magma *f*o_2_ (*20*). Numerous works have suggested that oxidized S is transferred from subducting slabs to the mantle wedge (*21*–*28*), yet debate remains concerning the importance of S as an oxidant in the mantle under arcs (*29*, *30*). How magmatic S contents relate to the concentration of S in the mantle wedge, to mantle redox, and to the concentration of other volatile elements or tracers of slab-mantle interaction remains less understood. The origin of slab-derived S continues to be debated: Previous publications suggested various oxidized hosts, such as seawater (*26*), sediments (*31*), and altered oceanic crust (AOC) (*25*) as sources of S in the subarc mantle wedge. Other studies suggest that the bulk slab undergoes redox reactions during devolatilization to produce fluids that convey more soluble oxidized S species to the mantle wedge (*23*, *28*, *32*, *33*).

The S isotopic composition (^34^S/^32^S, expressed as δ^34^S in a permille notation, ‰) of different lithologies in subducting slabs is highly variable and has the potential to trace the origin of S in the mantle wedge. Relative to the upper mantle, barite- and sulfide-rich sediments are enriched in ^34^S and ^32^S, respectively [between +20 and −45‰ (*34*)]. Similarly, the AOC contains anhydrite-rich layers with elevated δ^34^S and sulfide-rich lithologies with low δ^34^S values (*35*). Serpentinites have highly variable S contents (from <100 to >1000 μg/g), with S-rich serpentinites enriched in ^34^S (*36*). Despite this variability in subduction zone inputs, arc magmas exhibit a globally narrow range in δ^34^S values, between +1 and +7‰ (*25*–*27*). Correlations between δ^34^S and slab tracers [strontium (Sr)/neodymium (Nd) and lead (Pb)/cerium (Ce)] exist on an arc-to-arc basis (*25*, *26*); however, these correlations are less apparent on a global scale (*27*). Further data are necessary to better constrain the cause of elevated δ^34^S in the source of arc magmas relative to MORBs and the depleted MORB mantle (DMM).

In this work, we aim to provide constraints on the subduction zone S cycle. We use S isotope data collected from three arc systems with contrasting thermal structures (*37*), subducting slab age (*38*), and sediment inputs (investigated by various DSDP, ODP, and IODP cruises): the Aleutian, the Mariana, and Tonga arcs. We carry out a detailed investigation of degassing processes that affect the δ^34^S value of melts and gases, with important global implications for the S isotopic composition of volcanic gases that are measured at the surface. Using our data, we derive the S content and δ^34^S value of mantle-derived arc melts. We combine our data with existing δ^34^S values from three other arcs (Central America, Kyushu, and Cascades), alongside literature S, trace element, and redox data from individual volcanoes representing six additional arcs. Using these data, alongside mantle melting and binary mixing models, we connect magmatic S contents and δ^34^S values to the subarc mantle wedge on a global scale and emphasize the importance of S transfer between subducting slabs and the upper mantle on redox exchange between the surface and the deep mantle.

## RESULTS

**Fig. 1.**
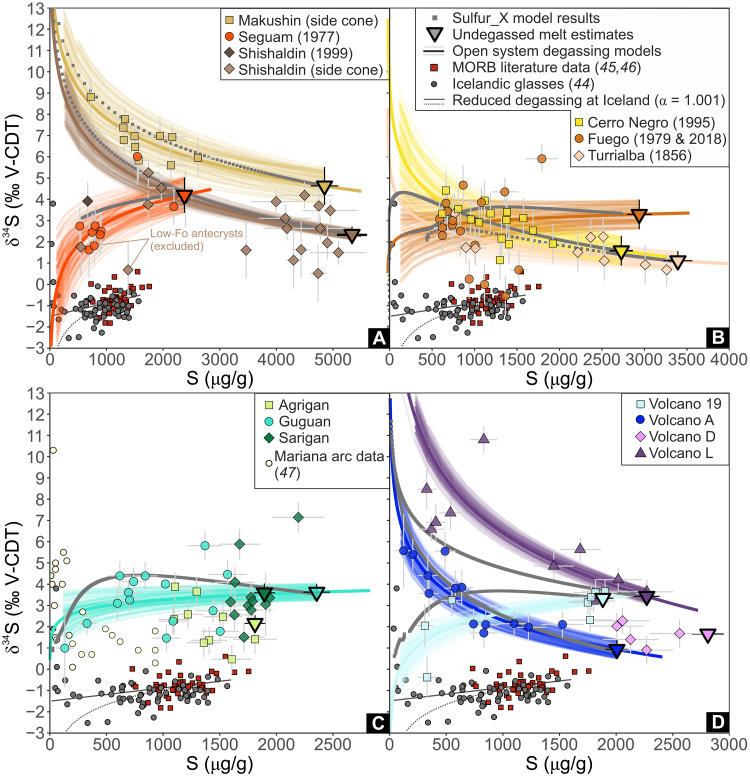
S isotope ratios plotted versus S content. Each panel represents a different volcanic arc: Aleutian Islands (**A**); Central America (**B**), including data published by Taracsák *et al.* (*27*); Mariana Islands (**C**), and Tonga (**D**). For localities with a degassing signature (i.e., where the lowest S content measured in a glass sample was below 70% of the maximum S content), the undegassed melt δ^34^S was estimated using open system equilibrium degassing models (thick lines), calculated using a regression approach (*27*). Thin lines show 100 degassing paths calculated by randomly sampling the 1 SE intervals for both the α_(gas-melt)_ value and the undegassed melt δ^34^S estimate. Gray, filled squares, some of which appear as lines because of data density, are forward degassing models calculated using version 1.2 of Sulfur_X (*51*). Model input parameters for the Sulfur_X calculations are provided in file S1. For localities where measured MIs underwent less than 30% S degassing (i.e., Sarigan and Volcano D), no degassing correction is applied. Instead, the average of all measured MIs is used as the undegassed melt δ^34^S estimate. Dark gray circles are subglacial glasses and whole rocks from Iceland (*44*), while the gray solid and dashed lines are closed and open system degassing curves, assuming an initial δ^34^S of −0.5‰, a α(gas-melt) of 1.001, and a starting S content of 1600 μg/g. Red squares are global MORB data from Labidi *et al.* (*45*, *46*). Light yellow circles in (C) are submarine and subaerial whole-rock data from the Northern Mariana arc [(*47*); excluding Mariana Trough back arc data]. Error bars are 1σ.

We report ^34^S/^32^S ratios from 158 volcanic glasses, including melt inclusions (MIs), melt embayments, and matrix glasses (Fig. 1)—these analyses include data (36 analyses) originally published by Taracsák *et al.* (*27*) from the Central American volcanic arc. Data were collected from 15 samples, representing 13 volcanic systems and 4 arcs (Central America, Aleutians, Marianas, and Tonga). Furthermore, we analyzed volatile contents, including hydrogen (H), carbon (C), chlorine (Cl), fluorine (F), and S, and concentrations of boron (B) and lithium (Li) via secondary ion mass spectrometry from the same glasses. Major element contents from the glasses were measured using electron probe microanalyses (EPMAs). All data are reported in file S1. All the studied samples were subject to previous MI studies—details on previous work are provided in Materials and Methods. We note that data points originally published by Taracsák *et al.* (*27*) that contained less than 500 μg/g S have been omitted from our dataset because of the limitations of the calibration standards used in that study (see Materials and Methods for more details).

On the basis of their measured silica and alkali contents (i.e., without applying any corrections for postentrapment iron (Fe) diffusion or crystallization/melting to MI data), the studied glasses are dominantly of basaltic composition (*39*). At some localities (Fuego and Turrialba from Central America), the melt reaches basaltic-trachyandesite and trachyandesite compositions. Olivine-hosted MIs are found in crystals containing between 57 mol % (Shishaldin 1999) and 92 mol % (Volcano L) forsterite. Volcano A and Volcano L from Tonga have high silicon dioxide (SiO_2_) and high magnesium oxide (MgO) contents and are therefore considered boninites (*40*). Concentrations of titanium dioxide (TiO_2_) vary between different samples, with some Tonga and Mariana samples depleted in TiO_2_ (<1 wt %) and other localities having elevated TiO_2_ (>2.0 wt %; Turrialba 1856 eruption, Shishaldin 1999 eruption, fig. S1). Sodium oxide (Na_2_O) contents vary from <1.5 wt % (Volcano A and Volcano L) to >5.0 wt % in evolved Fuego glasses. Total iron contents measured in glasses range from 4.9 to 15.4 wt % in our datasets; low-Fe glasses were measured in Volcano D and Volcano L samples from Tonga and Virgin cone, Shishaldin. We find variable relationships between Fe and S contents at different localities: Only Fuego glasses show a broad positive correlation, which may indicate sulfide saturation (fig. S2).

Li and B contents in the glasses are between 2.2 and 20 μg/g and between 2.3 and 46 μg/g, respectively. The highest Li and B contents were measured in Seguam 1977 and lowest in Tonga arc glasses (fig. S6). Ratios of B/Li vary from ∼0.5 at Turrialba to ∼6.7 at Volcano A in Tonga. Water contents in MIs generally fall within the 2 to 5 wt % range, overlapping with the global arc average of 3.9 +/− 0.4 wt % (fig. S4) (*41*). A notable exception is Makushin, with <1 wt % H_2_O in MIs. Previous MI data measured in the same sample indicate H_2_O contents up to 3.4 wt % (*42*). The low H_2_O contents measured in our MIs are likely a result of diffusive H loss (*43*). Concentrations of carbon dioxide (CO_2_) vary from below the detection limit at Volcano L to >3000 μg/g at Shishaldin (Virgin cone) and Seguam. As for other incompatible elements, Turrialba and Shishaldin 1999 melts are enriched in F relative to other localities, with Turrialba magmas reaching >2000 μg/g F content. At other localities, F contents are generally below 1000 μg/g. Overall, F correlates well with melt Cl contents (which vary from 300 to 2500 μg/g; fig. S5); this correlation indicates that the MIs were not contaminated by seawater on the seafloor (in the case of submarine Tonga samples) or in the shallow magmatic system, as seawater addition is expected to increase melt Cl concentrations at near-constant F content.

Arc magmas are known to contain considerably more S than MORBs (*20*). In our dataset, the maximum measured S content at each locality is the lowest at Agrigan (1600 μg/g) and the highest (up to 5600 μg/g) in MIs from Shishaldin (Virgin cone). S contents are highly variable in most samples because of S being partially degassed—this is indicated by correlations between H_2_O and SiO_2_ and S content (figs. S3 and S4). Ratios of S/potassium (K) either show a decrease at constant potassium oxide (K_2_O), particularly in our least evolved MIs hosted in high–forsterite content (Fo) olivines (Shishaldin-Virgin cone and Makushin-Pakushin cone), or a negative correlation in our more evolved samples (Shishaldin 1999, Fuego). The former is expected because of S degassing alone, while the latter relationship is indicative of concurrent silicate crystallization and S degassing.

S isotope compositions (δ^34^S) of the glasses are between −0.5 and 10.1‰. Variable relationships can be observed between δ^34^S values and melt S contents at different localities, including a decrease in δ^34^S with S content (Seguam), uniform δ^34^S at different S contents (Shishaldin Virgin cone, Fuego) and an increase in δ^34^S with S content (Volcano A, Volcano L, Cerro Negro, and Makushin) (Fig. 1). Overall, δ^34^S values in arc glasses are higher than those measured in Icelandic subglacial glasses (*44*) and in submarine MORB samples (Fig. 1) (*45*, *46*), which agrees with previous analyses from the Cascades (*25*) and Kyushu (*26*). Compared to previous whole-rock data from subaerial and submarine glasses from the Northern Mariana Arc (*47*), our Mariana arc MI data have considerably higher S contents (between 1000 and 2000 μg/g compared to <300 μg/g). On the other hand, considering the difference in S content, the two datasets have similar δ^34^S ranges: Whole-rock data are between +0.1 and +10.3‰ (10 of 11 data points are between +0.1 and +5.5‰), while Mariana MIs with >1000 μg/g S have δ^34^S values between +0.5 and +7.1‰ (Fig. 1C).

## DISCUSSION

### Tracking S degassing in arc magmas using δ^34^S

S is volatile and degasses from the melt in response to the pressure decrease. Degassing of S from silicate melts is a complex process: It may be present as sulfur dioxide (SO_2_) and hydrogen sulfide (H_2_S) [and minor carbonyl sulfide (OCS)] in the gas phase (*48*) and as dissolved sulfide and sulfate ions in the melt. The speciation of S in melts is primarily influenced by temperature, pressure, and *f*o_2_ (*13**–**15*, *19*, *49*), while the most important parameters that influence the speciation of S in the gas phase are *f*h_2_o [hence, pressure (*50*)], *f*o_2_, and temperature (*48*, *51*). On the basis of empirical parameterizations, S degassing can be modeled using two major degassing reactions (*51*)

$${\text{FeS}}_{\text{melt}}+H_{2}O_{\text{fluid}}\to H_{2}S_{\text{fluid}}+{\text{FeO}}_{\text{melt}}$$ (1)

$${\text{CaSO}}_{4,\text{melt}}\to {\text{SO}}_{2,\text{fluid}}+0.{\text{5O}}_{2,\text{fluid}}+{\text{CaO}}_{\text{melt}}$$ (2)

**Fig. 2.**
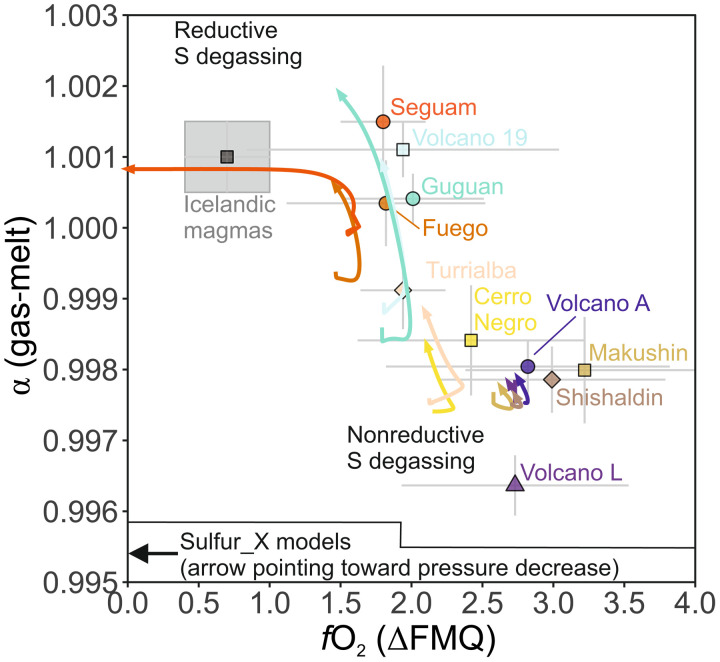
Gas-melt fractionation factors calculated from the regression models plotted against *f***o**_2_. Symbols are the same as in Fig. 1 for the studied volcanoes. Regression models are also presented in Fig. 1. For each location, average *f*o_2_ was used from all available data, where more than one data point was available. For Seguam, S speciation data from Zimmer *et al.* (*4*) were converted to *f*o_2_ using the model of O’Neill and Mavrogenes (*14*). Iron speciation data from Guguan (*63*) were converted to *f*o_2_ using the model of O’Neill *et al.* (*60*). For Icelandic magmas (gray box), Fe^3+^/ΣFe (0.15 to 0.20) are taken from Shorttle *et al.* (Reykjanes Ridge) (*133*) and Hartley *et al.* (Laki 1783 eruption) (*67*), while S isotope gas-melt fractionation factors are from Ranta *et al.* (*44*). Solid lines are the results of degassing models calculated using Sulfur_X (*51*), using the same starting parameters as in Fig. 1 (see file S1). Forward model lines are restricted to only show degassing paths for the S content range captured by our MIs. Arrows on the curves indicate the change in α_g-m_ and *f*o_2_ with pressure decrease. Error bars are 1 SEs for modeled α(gas-melt) values, as these were derived via regression, and 1 standard deviation for *f*o_2_ (plotted as ΔFMQ, the relative difference to the fayalite-magnetite-quartz buffer in log_10_ units).

Reactions 1 and 2 are dominant in reduced and oxidized magmas, respectively. Crucially, S degassing may drive the S isotopic composition of the melts and fluids in different directions depending on the speciation of S in the melt and the fluid phase (*52*, *53*). Gas-melt S isotope fractionation factors [α_g-m_, calculated as (^34^S/^32^S_gas_)/(^34^S/^32^S_melt_)] are known to vary as a function of melt redox state because of its influence on melt S speciation (*53*, *54*). In practical terms, degassing of S from a reduced magma (where S is present mainly as dissolved sulfide) is expected to drive S isotope ratios in the melt toward negative values because α_g-m_ is larger than 1, while the opposite is true for oxidized melts, for which α_g-m_ is smaller than 1 (Figs. 1 and 2). Therefore, trends in melt S isotopic composition as S is degassed can be used to track which reaction is dominant during degassing and provide information on the storage depth and melt redox during gas exsolution (*55*). However, these degassing trends may obscure the mantle-derived S isotopic signature of magmas (Fig. 1).

Fractionation factors can be estimated using our δ^34^S data for localities that show sufficient degassing signals. We find substantial S degassing in 9 of 12 studied localities. We did not estimate fractionation factors for Volcano D, Agrigan, and Sarigan. At these localities, all analyzed glasses had S contents that were less than 30% degassed compared to the maximum measured S content, i.e., had insufficient degassing signals to carry out model calculations. We estimate fractionation factors using the regression method described by Taracsák *et al.* (*27*), assuming equilibrium open system degassing (Fig. 1). Briefly, this involves fitting regression between the fraction of remaining S in the melt (derived from melt S content) and the S isotopic composition of the melt and using the slope of the regression to calculate α_g-m_.

The results of the regression models, alongside errors estimated using Monte Carlo modeling, are presented in Fig. 1, while the median α_g-m_ values are shown as a function of *f*o_2_ in Fig. 2. Six volcanoes (Cerro Negro, Turrialba, Makushin, Shishaldin-Virgin side cone, Volcano A, and Volcano L) show an increase in δ^34^S with decreasing S content. S isotope ratios show no correlation with S content in Guguan and Fuego glasses. At Fuego, there is considerable spread in δ^34^S values at given S content, and as a result, the degassing trend of Fuego is the least defined of the studied localities. We note that on the basis of our forward degassing models, Fuego magmas fall within an *f*o_2_ range where even small changes may lead to α_g-m_ changing from below 1 to above 1, which may drive such a spread in MI S isotope values. S isotope ratios decrease with S content at Volcano 19 and at Seguam. While the predicted fractionation factors for Central American volcanoes originally analyzed by Taracsák *et al.* (*27*) have changed—increasing by 0.0013 for Fuego and decreasing by 0.0006 and 0.0009 for Cerro Negro and Turrialba, respectively—the revised estimates remain within the error of those previously published. This change is due to the exclusion of matrix glasses and MIs with low S contents (<500 μg/g) from our dataset, which had a strong leverage on the regressions presented by Taracsák *et al.* (*27*) but, as subsequently determined, also had large errors because of both analytical conditions and data correction procedures. We excluded two MIs from our regression model for Shishaldin (Virgin cone) that were hosted in low-Fo (72 mol %) olivines, as we interpreted these as unrelated to the carrier magma (which was less evolved and contains olivines with Fo above 88 mol %).

Sulfide fractionation can also influence δ^34^S measured in glasses (*11*, *17*, *56*, *57*). Using the equations presented by Marini *et al.* (*17*) and assuming melt S^6+^/ΣS between 0.5 and 0, we estimate that α_sulfide-melt_ at 1000°C is between 0.9984 and 1.0006, while at 1200°C, it is between 0.9988 and 1.0004. Therefore, under reducing conditions, sulfide removal from the silicate melt causes a small decrease in melt δ^34^S, while at a higher melt sulfate fraction, sulfide removal increases melt δ^34^S. Therefore, sulfide fractionation has a similar effect to degassing (Fig. 2). As both processes result in a similar relationship between S and S isotopes ratios, we cannot fully rule out a minor role for sulfide removal for Fuego and Seguam samples, albeit the effect of this on δ^34^S is likely small (at most −1.1‰, similar to our analytical uncertainty) at low melt S^6+^/ΣS (<20% of the original S from the melt lost to sulfide). While substantial sulfide removal at high melt S^6+^/ΣS could theoretically cause the trends observed in our more oxidized systems (Volcano A, Volcano L, Makushin, and Shishaldin), high melt S^6+^/ΣS inherently results in low melt S^2−^ contents, making this process unlikely. Another potential indicator of sulfide removal is a positive correlation between the Fe and S concentrations in glasses—this correlation would suggest a crystallization-driven decrease in S concentration at sulfide saturation (SCSS) because of lower melt Fe content and temperature and consequent sulfide saturation. Such a relationship between total FeO and S content is observed for Fuego glasses but not in other systems (fig. S2). We did not find petrological evidence for sulfide saturation in any of the studied systems, such as sulfides in the tephra groundmass or olivine- and clinopyroxene-hosted sulfide inclusions. It is possible that the absence of sulfides in the matrix is due to sulfide resorption/breakdown resulting from S degassing at shallower depths (*58*)—this process may also cause further scatter in S-δ^34^S data. While a few MIs in some samples contain sulfide droplets (e.g., Agrigan), considering the lack of evidence for sulfide saturation in the carrier magma, these are likely formed because of postentrapment processes, including melt reduction–driven sulfide saturation (*59*). MIs containing sulfides were excluded from our dataset. On the basis of the above arguments, we conclude that the most likely explanation for differences in the relationship between δ^34^S and melt S content at various localities is due to variable conditions, particularly the melt redox state, during magma degassing.

Using our regression modeling approach, we calculate that α_g-m_ values during degassing of arc magmas are between 0.9963 ± 0.004 at Volcano L and 1.0015 ± 0.007 (1 SE) at Seguam (Fig. 2). We observe a decrease in α_g-m_ with increasing melt *f*o_2_ (Fig. 2; see the Supplementary Materials for details on how *f*o_2_ and Fe and S speciation were estimated for each system). To explain this shift in α_g-m_ as a function of *f*o_2_, we modeled fractionation factors expected during magmatic degassing for each system using version 1.2 of Sulfur_X (*51*). In summary, this involves first calculating degassing pathways for a C-H-S–containing melt and fluid phase at a constant temperature, assuming a starting H_2_O, S, and CO_2_ content for the melt (see file S1 for input parameters for each volcano). Sulfur_X calculates melt and fluid S speciation as a function of oxygen and volatile (H_2_O and CO_2_) fugacities. S isotope fractionation factors during degassing are calculated using modeled gas and melt S speciation and the equations presented by Marini *et al.* (*17*). As degassing progresses, α_g-m_ changes because of concurrent variation in both melt S speciation, hence *f*o_2_ (*13*, *14*), and gas S speciation (SO_2_/H_2_S), which is a function of *f*o_2_ and *f*h_2_o [and, consequently, pressure (*53*)]. In our model, the largest α_g-m_ values can be explained if a comparatively reduced melt is degassing S as H_2_S (resulting in an α_g-m_ of ∼1.001, as modeled for Seguam using Sulfur_X) or mainly SO_2_ (α_g-m_ between 1.002 and 1.003, as calculated for Guguan during the final stages of degassing; Fig. 2). The lowest α_g-m_ values (such as calculated for Volcano L via regression) can be achieved by degassing sulfate from the melt as H_2_S (*53*), but such a degassing reaction is not predicted to be a major process in magmatic systems (*51*). None of our Sulfur_X models predict this to be a dominant process: All oxidized volcanoes in our dataset are predicted to degas a SO_2_-rich fluid (fig. S9), resulting in α_g-m_ values near 0.998 (Fig. 2). At Makushin, our Sulfur_X model calculations predict α_g-m_ values down to 0.996 but only for the final stages of degassing between 2 to 6 MPa pressure, corresponding to near-surface conditions (fig. S9). On the basis of our modeled α_g-m_ values, arc magmas have *f*o_2_ values that fall within the narrow (∼2 log units) interval in which the sulfide-to-sulfate transition occurs within silicate melts. As a result, more reduced arc magmas degas S from dissolved sulfide (such as Seguam and Volcano 19), while more oxidized arc magmas degas S from dissolved sulfate (Cerro Negro, Volcano A, and Makushin; Fig. 2). On the basis of our α_g-m_ estimates, the shift from low to high S^6+^/∑S values in arc melts occurs as they reach an *f*o_2_ value that is 1.5 log_10_ units above the fayalite-magnetite-quartz buffer (ΔFMQ+1.5, corresponding to an Fe^3+^/ΣFe of >0.2). In our calculations, we convert between S^6+^/∑S, Fe^3+^/ΣFe, and *f*o_2_ using the equations of O’Neill *et al.* (*60*) and O’Neill and Mavrogenes (*14*), while many previous publications use an older equation (*61*) to convert between Fe^3+^/ΣFe and *f*o_2_, which is based on a different calibration dataset and, as a result, calculates a lower *f*o_2_ estimate (in the range relevant to modern arc magmas) at a given Fe^3+^/ΣFe (*62*). Because of this, our *f*o_2_ estimates are higher than many literature values for the same volcanoes [e.g., Marianas (*63*)]. Our independent *f*o_2_ estimates are also somewhat higher, often above FMQ+2, and reaching FMQ+3 at Shishaldin and Makushin for volcanoes where *f*o_2_ was estimated using V partitioning between olivine and melt using the equation of Shishkina *et al.* (*64*), whose calibration dataset is optimized for hydrous arc magmas. It has been suggested that some (<1 log unit) bias exists for most V partitioning calibrations available in the literature (*65*), which may result in overestimation of *f*o_2_ for colder, hydrous melts; this bias is the smallest when using the calibration of Shishkina *et al.* (*64*) and Erdmann *et al.* (*65*), which match for our dataset within 0.15 log units.

Our degassing models also track the *f*o_2_ change as a function of S loss from the melt: At more reduced volcanoes (Fuego, Guguan, Seguam, and Volcano 19), *f*o_2_ decreases by 0.5 to >1 log units in response to S degassing (Fig. 2). Such S degassing–driven melt reduction has been proposed to be a globally prevalent process in arc magmas (*66*). This process has been recorded in various geodynamic settings, such as intraplate and mid-ocean ridge systems, including Laki in Iceland (*67*), Erebus (*68*), Hawaii (*69*, *70*), and the Canary Islands (*71*). However, these observations are based on data collected from volcanic systems where Fe^3+^/ΣFe is between 0.1 and 0.25 (*f*o_2_ below FMQ+1.5). This range does not reflect the total redox variability found in arc volcanic rocks where Fe^3+^/ΣFe can reach >0.3, as demonstrated by data collected from Augustine in Alaska (*72*), Aoba (Vanuatu), and Vulcano in the Aeolian Arc (*73*). Using an independent constraint, i.e., S isotope fractionation during degassing, we argue that in more oxidized systems (>FMQ+2 or melt Fe^3+^/ΣFe above 0.25), substantial reduction of the melt via S degassing is not expected. Such oxidized systems are represented in our dataset by primitive scoria samples from the Aleutian arc (Virgin cone, Shishaldin, and Pakushin cone, Makushin), submarine Volcano A and Volcano L in the Tonga arc, and Cerro Negro in the Central American arc (Figs. 1 and 2). This interpretation is supported by our Sulfur_X degassing models (Fig. 2) and agrees with experiments and thermodynamic models that predict little *f*o_2_ change, or even oxidation, in response to S degassing in oxidized magmas (*49*, *74*).

Regardless of these caveats, Fig. 2 illustrates the expected correlation between gas-melt S isotope fractionation factors and arc magma *f*o_2_ using completely independent datasets (δ^34^S-S data in MIs on the one hand and magma redox proxies on the other). This is directly manifested in the great variety of degassing trends shown in Fig. 1, whereby more oxidized melts have increasing δ^34^S during degassing, while more reduced melts decrease. Therefore, the δ^34^S compositions of volcanic SO_2_ and H_2_S measured in gases at the surface, or in highly degassed whole rocks, may be affected by fractionation toward either positive or negative values or not at all. Using our data from nine volcanic systems, we estimate equilibrium gas δ^34^S values between −1 and +4.5‰ upon degassing initiation. After extensive degassing (95%), δ^34^S values increase, with eight of the nine studied systems falling between +1 and +5‰, apart from Volcano L from Tonga (+11‰). This predicted δ^34^S range overlaps the range in gas data collected from various arc volcanoes globally, which is between −2 and +13.5‰ based on compiled global data (*25*). Our results demonstrate that in most cases, correction for degassing-driven isotope fractionation is necessary to investigate the S isotopic composition of undegassed arc magmas. As the δ^34^S of magmatic fluids may change during magma ascent, volcanic gas δ^34^S values measured at the surface may be used to track changes in degassing depth beneath volcanic systems. Therefore, gas δ^34^S data can be a useful tool for volcano monitoring, once the δ^34^S evolution of the melt has been established through the analyses of MIs, bulk tephra samples, and matrix glasses, and the depth dependence of the degassing magma determined using a model such as Sulfur_X (*51*) or VolFe (*75*).

### Redox, trace elements, and S contents in arc magmas: A global view

**Fig. 3.**
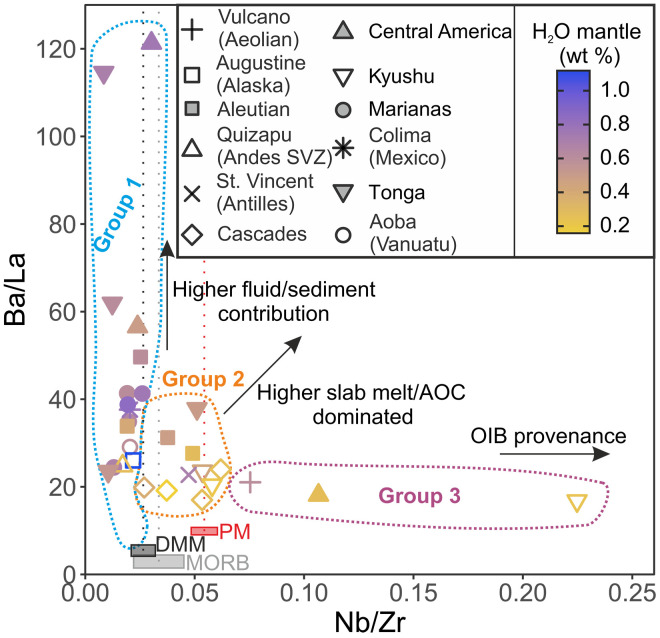
Average Ba/La versus Nb/Zr ratios of the 37 arc volcanic systems included in our model calculations. Trace element compositions for each locality are based on whole-rock and MI compositions later used for melting degree estimation. Where trace element analyses from whole-rock samples are not available, we use the average of MIs from which *f*o_2_ and maximum S content are taken. Symbol colors are based on the H_2_O content of the mantle source, calculated for each locality using *D*_0_ (bulk partition coefficient) and *P*_0_ (partition coefficient weighted by the melting fraction of each mineral phase in the mantle) values of 0.0122 and 0.0189, respectively. Melting degrees used to calculate mantle H_2_O contents are presented in Fig. 5. Trace element composition of the DMM, MORB, and primitive mantle (PM) are from Salters and Stracke (*81*), Hofmann (*134*), and Palme and O’Neill (*135*), respectively. Dotted vertical lines show the average Nb/Zr ratio of DMM, MORB, and PM. The arrow pointing right indicates the expected compositional vector for localities with ocean island basalt (OIB) provenance.

While arc magmas sample various mantle sources globally, reflected in their highly variable trace element compositions (Fig. 3), they are, in most cases, more oxidized than MORBs (*25*, *63*, *73*, *76*, *77*), which means that they have increased S solubility because of the presence of dissolved S^6+^ (*14*). As a result, S contents in arc magmas have been used to argue that a slab component added to the mantle wedge drives simultaneous oxidation and S enrichment in arc magmas (*20*). Similar to the approach of Muth and Wallace (*20*), we compiled a global dataset of arc magma compositions to investigate the relationship between S, other volatiles, trace elements, and mantle redox. However, our approach has a number of fundamental differences compared to the model of Muth and Wallace (*20*), whose goal was to provide conservative estimates on arc magma and mantle wedge S contents. For our data compilation, we only include localities that have data from which an independent estimate for melt *f*o_2_ can be derived. We also limit our dataset to localities for which trace element and magmatic H_2_O contents are available and to samples that have MgO contents >5 wt %. These criteria are necessary to exclude any melt compositions that crystallized large amounts of clinopyroxene or plagioclase, which makes primary melt composition estimates unreliable (and results in erroneous melting pressures and temperatures), as existing models only use olivine addition/subtraction to correct melt composition (*78*, *79*). To estimate the undegassed H_2_O content of arc melts, we take the highest H_2_O concentration measured from each locality to limit the potential effect of degassing and diffusive H_2_O loss as much as feasible, as both of these processes would cause underestimation of H_2_O contents. Of these criteria, the main limiting factor is the existence of independent *f*o_2_ estimates. In total, we compiled data from 29 arc volcanoes that matched our criteria (Fig. 3). These data include 102 whole-rock and glass (MI) analyses and encompass all the volcanic systems with δ^34^S data presented here. Of these 102 compositions, we calculated realistic melting temperature and pressure (i.e., excluding values where melting pressure corresponded to crustal and not mantle depths) for 93 in total. Input data and further details on model calculations are provided in file S1.

The trace element variability in the 37 selected volcanic systems (in some cases, multiple samples came from the same volcano but from different eruptions/parasitic cones) is illustrated using niobium (Nb)/zirconium (Zr) and barium (Ba)/lanthanum (La) ratios (Fig. 3). Ba/La is widely used as a slab tracer (*80*), while Nb/Zr ratios are highly sensitive to both the melting degree, the presence of recycled mantle components (e.g., pyroxenite), and previous melt removal events in the mantle source of magmas. Using these trace element ratios, we categorized the studied systems into three groups. Group 1 has low Nb/Zr [≤DMM estimates (*81*)] and highly variable Ba/La contents, ranging from <10 (Cascades) to >100 (at Cerro Negro, Central America and Volcano A, Tonga). These localities are likely influenced by a sediment- and fluid-dominated slab component, which preferentially transferred fluid-mobile elements like Ba into the mantle relative to melt-mobile incompatible elements like Nb. This interpretation is evidenced by the elevated mantle source H_2_O contents of group 1 localities (Fig. 3). Group 2 has elevated Ba/La and higher Nb/Zr than DMM, indicating that both Nb and Ba were added to the mantle source; we interpret this to indicate the presence of a larger slab melt component, including the AOC melt (*82*). Group 3 comprises three localities (Turrialba, Vulcano, and Fukue-Onidake) with Nb/Zr ratios above those explained by peridotitic mantle lithologies and likely require a recycled crustal lithology in their source either directly from the slab or in the form of pyroxenite that was present in the mantle before the onset of subduction.

**Fig. 4.**
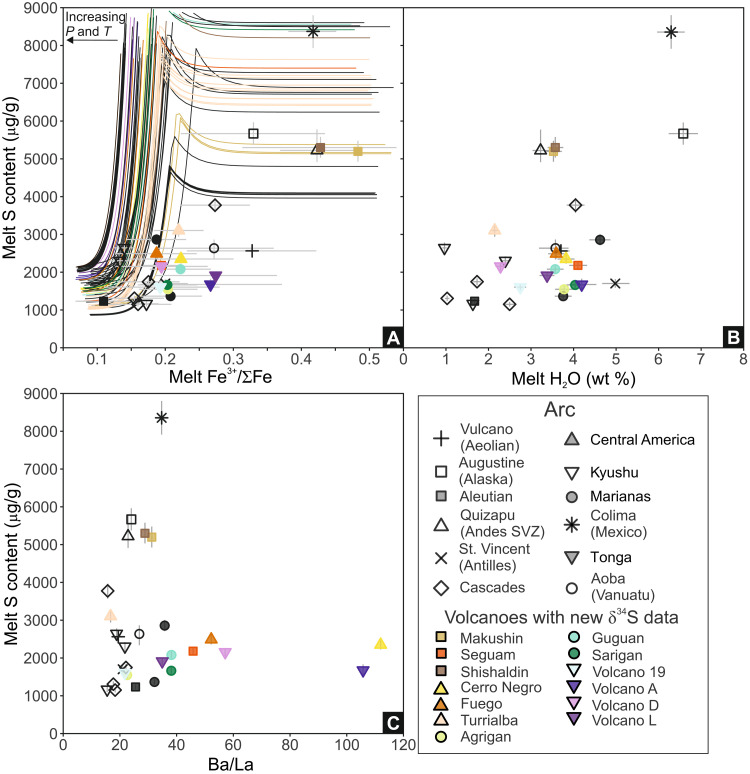
S content estimates for arc primary melts plotted against various geochemical proxies. S content is plotted against primary melt Fe^3+^/ΣFe (**A**), primary melt H_2_O content (**B**), and measured Ba/La ratio (**C**). Symbols for each arc are the same as in Fig. 3—colors of symbols are from Fig. 1 for localities with δ^34^S data presented in this work. Symbols represent the median value, while error bars show a 70% (15 to 85%) interval of the probability distributions calculated using Monte Carlo simulation. Olivine fractionation correction was carried out using the method of Lee *et al.* (*78*) using variable H_2_O and Fe^3+^/∑Fe contents—uncorrected and corrected data are provided in file S1. Lines in (A) show modeled melt S solubility as a function of Fe^3+^/ΣFe. These lines were calculated for all 93 primitive melt compositions by calculating S^6+^/ΣS values using the model of O’Neill and Mavrogenes (*14*), while S content at sulfide and anhydrite saturation (SCSS and SCAS) was calculated using the models of Fortin *et al.* (*107*) and Zajacz and Tsay (*108*), respectively. Colors of the S solubility curve correspond to symbol color (i.e., gray if no δ^34^S data are available for locality). From some arcs, only one volcano is included—for these arcs, the volcano name is given first, and the name of the arc is provided in parentheses. The arrow in the top left corner of (A) indicates that the direction S solubility curves would move toward in case of increased pressure and temperature.

Estimates of redox (expressed as both *f*o_2_ and melt Fe^3+^/∑Fe) from arc systems are calculated using different methods (Fig. 2): olivine-melt vanadium partitioning, converted to *f*o_2_ using the calibration of Shishkina *et al.* (*64*); direct measurements (not corrected for postentrapment crystallization and Fe diffusion) of Fe speciation from glasses measured by x-ray near edge spectroscopy, converted to *f*o_2_ using the method of O’Neill *et al.* (*60*); olivine-spinel oxybarometry, converted to *f*o_2_ using the equation of Ballhaus (*83*); and EPMA-based S speciation estimates (including Seguam and Kyushu samples), converted to *f*o_2_ using the equation of O’Neill and Mavrogenes (*14*). A temperature estimate is required to convert log(*f*o_2_) to a ΔFMQ notation and to melt Fe^3+^/∑Fe values. We use equilibrium between MI-olivine pairs to estimate temperature using the thermometer of Putirka (*84*) optimized for H_2_O-rich melts. Estimates of *f*o_2_ for the studied system vary from FMQ+1 for the 1999 eruption of Shishaldin (Aleutians) to FMQ+3 at Pakushin cone, Makushin (Aleutians), and at Cerro Azul/Quizapu (Los Hornitos cone, Southern Volcanic Zone, Andes). This variability in *f*o_2_ is demonstrated by the large Fe^3+^/ΣFe (0.11 to 0.48) of the studied systems (Fig. 4A). Some of our estimates are higher by up to 1 log unit than literature estimates from the same localities. For example, for the Los Hornitos cone, Tassara *et al.* (*77*) estimate *f*o_2_ near FMQ+2.6, while our estimate is FMQ+3.2. These differences are due to the use of uncorrected Fe^3+^/ΣFe values and a different conversion method (see fig. S10B). All references for data sources and *f*o_2_ estimates are provided in file S1.

At each locality, we estimate undegassed melt S content using maximum measured S contents. This approach can overestimate melt S contents because silicate fractionation increases melt S content in the absence of S degassing. This process would result in near-constant S/K at increasing melt K_2_O content. However, S/K in our samples either decreases at constant K_2_O content because of degassing or has a negative correlation with K_2_O because of concurrent fractionation and degassing, which validates our approach (fig. S4). This interpretation is also supported by negative correlations between S and SiO_2_ contents (fig. S3). Undegassed melt S estimates vary from 1500 μg/g at cone BBL, Lassen volcanic complex (Cascades), to 7200 μg/g at Colima, Mexico. These values are corrected for olivine fractionation or accumulation to derive the primary melt S content in equilibrium with the mantle wedge, which is between 1150 and 8360 μg/g at cone BBL, Lassen, and at Colima, Mexico, respectively.

Our estimates of primary melt S concentrations correlate with melt Fe^3+^/∑Fe estimates (Fig. 4A) and, hence, *f*o_2_. Furthermore, localities with high S contents also have overall high H_2_O concentration estimates, albeit this relation is more scattered (Fig. 4B). These findings are overall in line with those presented by Muth and Wallace (*20*), suggesting a causal relationship between melt redox and S content in arc magmas on a global scale. However, we find no correlation between traditional slab tracers, such as Ba/La (Fig. 4C), Pb/Ce, or Sr/Nd, and primary S concentrations—this may imply that if any S is added to the mantle wedge, it may not originate from the same lithologies as Ba, Pb, and Sr, which are sourced primarily from subducting sediments [Ba and Pb (*85*, *86*)] and the AOC [Sr (*82*)].

### Inverting primary arc magma compositions to those in the mantle wedge

Mantle melting can overwrite the relationship between volatiles, redox, and trace elements resulting from differences in partitioning during the melting process. Therefore, to better constrain relationships between redox, S, and H_2_O contents in subduction zones, we next consider variations in the mantle wedge. Modeling of the melting process in the mantle wedge is challenging: The addition of H_2_O from subducting slabs means that melting begins at a lower temperature and higher pressure than in nominally anhydrous mantle lithologies (*1**–**3*, *87*, *88*). The traditional approach of using trace elements to calculate the melting degree is complicated in subduction settings because of several potentially superimposed processes: (i) the addition of various melt- and fluid-mobile trace elements [light rare earth elements, thorium (Th), Ba, rubidium (Rb), Sr, and K] into the mantle wedge (*82*, *85*, *86*, *89*), (ii) trace element depletion through melting of the asthenosphere in back arcs before reaching the wedge corner (*40*, *90*), (iii) lithophile trace element enrichment in parts of the subducting igneous oceanic crust (*91*), and (iv) mantle heterogeneity predating subduction (*92*). These processes together cause variable trace element signatures in arc magmas (Fig. 3), driving the composition of the mantle wedge away from the prevalent DMM composition observed at ocean ridges.

#### Calculating mantle melt fraction

The major element compositions of magmas can be used to estimate equilibrium temperatures and pressures during melting (*78*) and, in turn, converted into estimates for the fraction of melting (*F*) using a solidus (*87*, *93*) and a pressure-dependent *dT*/*dF* value while, at the same time, taking into account the cryoscopic effect of H_2_O in the mantle during melting (*2*). This approach was previously used to estimate melting degrees under the Tonga and Mariana arcs (*40*, *94*). We use the method of Cooper *et al.* (*40*) to estimate melting degrees for our global dataset of arc compositions—details of these calculations are provided in Supplementary Text.

A second, independent estimate of *F* is obtained using primary melt sodium (Na) contents for each sample, assuming nonmodal batch melting of spinel lherzolite (see Supplementary Text for further details on model calculations). Na is thought to be a useful proxy of melting under arcs on the basis of correlations between crustal thickness and normalized Na content of global arc segments (*95*, *96*).

**Fig. 5.**
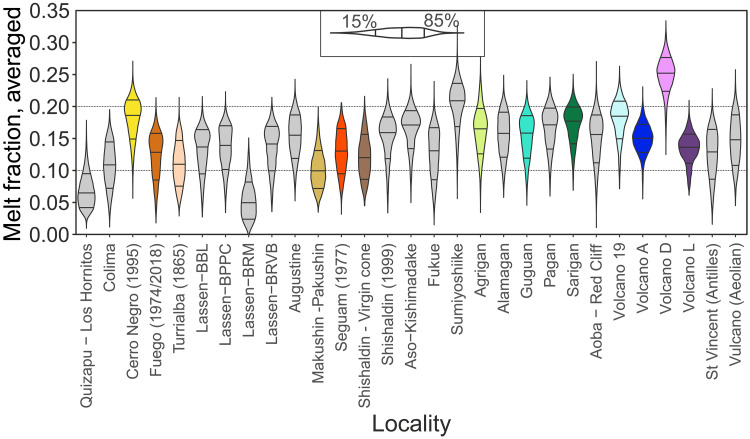
Averaged melt fraction estimates for selected arc systems on a violin plot. Each violin is based on the simulated data from our Monte Carlo model. Melting degrees plotted here are the average of the Na content–based and thermobarometry-based estimates. Lines within each distribution show the 15%, 50% (median), and 85% values. Horizontal dashed lines indicate the 10 to 20% melt fraction range, which we find is typical for many arc systems. Colors are based on Figs. 1 and 4.

The above-described estimates of *F* were averaged to calculate our final *F* values (93 primary melt compositions yield reasonable *F*), which are presented in Fig. 5. Errors associated with the different melting models were calculated using Monte Carlo simulations—the typical error for *F* is between ±0.02 and ±0.04 (expressed as melt fraction). Critically, both methods used to estimate *F* have their advantages and limitations. While the thermometer of Lee *et al.* (*78*) is based on a large calibration dataset including H_2_O-rich melts, it is mainly calibrated using low H_2_O and anhydrous compositions and does not consider CO_2_, which can strongly influence melt SiO_2_ content and skew estimated mantle melting pressure (*97*). In contrast, the Na-based model, which has an advantage in terms of simplicity, has a large uncertainty in terms of the mantle source Na content: Experimental data suggest that sediment and AOC melt can contain between 2.2 and 7.5 wt % NaO (*98*, *99*), which is an order of magnitude higher than the DMM estimate of 0.29 wt % (*81*). Therefore, substantial addition of sediment and AOC melt to the mantle wedge can result in higher Na contents in the source and underestimation of melting degrees. As a result, we opted not to favor one model over another.

**Fig. 6.**
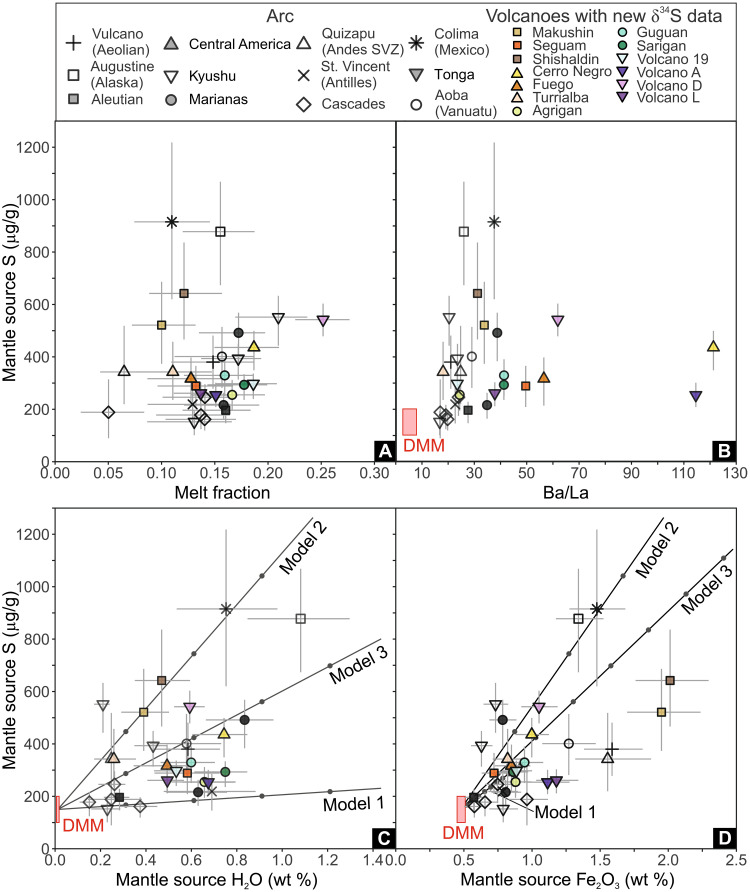
S contents in the mantle source of arc magmas. Median mantle wedge S contents are plotted against median melt fraction estimates (**A**), melt Ba/La ratios (**B**), and modeled H_2_O (**C**) and Fe_2_O_3_ (**D**) contents of the mantle wedge. In (C) and (D), three different mixing models are presented (models 1 to 3—see text for details) between a slab component [sediment melt from (*98*)] and DMM [trace elements and H_2_O from Salters and Stracke (*81*) and Fe_2_O_3_ from Davis and Cottrell (*104*)]. All three models assume 15 wt % H_2_O in the slab component (realistic for a fluid-saturated slab melt), while the S content and S and Fe speciation were varied between different models. Model 1 assumes an AOC/sediment melt containing dissolved sulfate and ferric iron and having low S capacity resulting from a small fluid component (0.1 wt % S, S^6+^/ΣS = 1, Fe^3+^/ΣFe = 1). Model 2 is a more reduced, fluid-rich slab component, with only two-thirds of S being oxidized (1.5 wt % S, S^6+^/ΣS = 0.66, Fe^3+^/ΣFe = 0.6), realistic for a fluid-melt mixture in equilibrium with AOC at <FMQ+1 (*33*). Model 3 assumes a fully oxidized fluid-melt mixture with intermediate S content, realistic for an oxidized AOC (>FMQ+1) fluid/melt mixture (0.7 wt % S, S^6+^/ΣS = 1, Fe^3+^/ΣFe = 0.6) that is more melt-rich (lower S capacity) than model 2. To calculate ferric iron contents of the mantle source, we assume that all S^6+^ reacts to S^2−^ in the mantle wedge before any melting under the arc, producing Fe^3+^. Circles represent 2% increments for the mixing models. Error bars are the same as defined in Fig. 5 (15 and 85% values taken from our Monte Carlo simulation).

*F* varies from 0.06 ± 0.03 for cone BBR at Lassen to 0.26 ± 0.02 for Volcano D, Tonga, with most (26 of 29) median *F* values falling between 0.1 and 0.2 (Fig. 5). Our melting degree estimates retain a relationship with crustal thickness (fig. S8) at arcs, as observed for global arc segment Na contents (*95*). Our global range for arc F overlaps with the range suggested in (*100*), which inferred melt fractions between 0.1 and 0.3 under arcs on the basis of fluid-immobile trace element systematics [titanium (Ti), Zr, and yttrium (Y)]. Overall, we consider that the mantle wedge undergoes larger degrees of melting than the mantle under ocean ridges, likely due to the combined cryoscopic effect of up to 1 wt % H_2_O present in the mantle source (Fig. 6) and various degrees of decompression controlled by the thickness of overlying crust (*101*, *102*).

#### Calculating S, H_2_O, and ferric iron in the mantle wedge

Using the estimates of *F* from Fig. 5, we inverted arc magma geochemistry (primary melt S and H_2_O contents) and redox [primary melt iron(III) oxide (Fe_2_O_3_)] to the composition of the mantle wedge. Using nonmodal batch melting models, we calculated mantle source H_2_O and ferric iron contents (Fig. 6) using partition coefficients taken from Rosenthal *et al.* (*103*) for H_2_O and Davis and Cottrell (*104*) for ferric iron (further details are provided in Supplementary Text). Mantle source S contents were calculated, assuming perfectly incompatible behavior for S during mantle melting. This assumption follows the same reasoning as presented in previous works (*27*, *105*, *106*): The overwhelming S budget of the mantle will be present in sulfide, and once all sulfide is consumed by melting, the total S budget of the mantle will be present in the silicate melt. This method calculates the absolute minimum mantle source S content: Higher mantle S contents are possible if sulfide is retained in the source. We test whether total sulfide removal is feasible for our studied volcanoes by calculating total S solubility (total dissolved S^2−^ and S^6+^) as a function of *f*o_2_ (Fig. 4A) for our 93 primary melt compositions. Using the total S solubility and *F* values, we derive an estimate of mantle S content at sulfide exhaustion for each composition. We then subtract these values from our mantle S content estimates (fig. S10A). If the resulting value is negative, we can assume total sulfide removal from the source. We find that most compositions in our dataset have mantle S contents below the estimated S content at sulfide exhaustion, apart from those from Los Hornitos cone, Cerro Azul/Quizapu (Chile), and from Colima, Mexico. Despite their high *f*o_2_ (>ΔFMQ+2.5), Los Hornitos and Colima magmas have a comparatively low S concentration at anhydrite saturation (SCAS) estimates at 4000 and 6900 μg/g, both of which are below our primary melt S content estimates (5200 and 8400 μg/g, respectively). However, these differences are overall minor: Both Quizapu and Colima primary melts fall close to (and within uncertainty) the boundary between S saturated and undersaturated melts (see fig. S9), indicating that minimal, if any, sulfide is retained in their source after melting. At both localities, the primary melt would be saturated with anhydrite (because of high *f*o_2_) and not sulfide. In summary, our findings differ from those of Muth and Wallace (*20*), who suggested that primary arc magmas remain sulfide-saturated during melting, which implies the presence of residual sulfide in the mantle wedge. Sulfide retention in the residual mantle wedge was also proposed on the basis of low platinum (Pt) contents measured in MIs from the Andes southern volcanic zone (*7*) and iridium (Ir)/palladium (Pd) ratios measured in sulfide grains from subduction-influenced mantle xenoliths (*22*). Large errors in some independent *f*o_2_ estimates (particularly those calculated using vanadium olivine-melt partitioning) introduce further uncertainty regarding sulfide exhaustion or retention in the mantle wedge. Any bias in the relative position of the sulfide-sulfate transition to *f*o_2_ can also move our primary melt Fe^3+^/ΣFe and S estimates toward the sulfide-saturated field in Fig. 4A. We note that the S speciation model of O’Neill and Mavrogenes (*14*) we use in Fig. 4A to calculate S solubility curves does not take pressure into account—higher pressures relevant to mantle melting is expected to push these curves to the left of the plot (*15*, *19*), away from our melt Fe^3+^/ΣFe and S estimates. Despite these caveats, our model calculations (fig. S10A) indicate either sulfide exhaustion or minimal sulfide retention in the mantle wedge.

Our modeled mantle wedge S contents vary between 160 and 920 μg/g, showing that at some localities, the mantle wedge contains little excess S relative to the upper mantle, while at other arcs, mantle wedge S contents are >6 times higher than the DMM range of 100 to 200 μg/g (Fig. 6). Mantle wedge S contents do not correlate with trace element ratios thought to be indicative of slab components in the mantle wedge, such as Ba/La (Fig. 6B). On the other hand, broad correlations between mantle S, H_2_O, and Fe_2_O_3_ contents (Fig. 6, C and D) indicate that H_2_O and S addition to the mantle wedge is accompanied by mantle oxidation. To explain modeled mantle wedge H_2_O, S, and Fe_2_O_3_ contents, we calculated two-component mixtures between DMM (*81*) and a slab component composed of a mixture of slab melt and fluid on the basis of experimentally produced sediment melt compositions from Hermann and Spandler (*98*). In our mixing models, we assume simultaneous mass flux from both fluid and melt. While slab components other than sediment melt can also contribute to the mantle wedge [such as melts from the AOC (*82*)], we find that using a sediment melt is sufficient to model H-S-Fe^3+^ systematics of slab-mantle interaction as oceanic crustal melts contain similar amounts of Fe as sediment melt (*98*, *99*). Furthermore, both sediment and oceanic crust melts have similar SCSS and SCAS values (*107*, *108*). The SCSS is below 700 μg/g for oceanic crust melt and <200 μg/g for sediment melts, while the SCAS is <2000 μg/g for both lithologies. For our model calculations, we assumed a DMM Fe_2_O_3_ concentration of 0.47 wt % and Fe^3+^/ΣFe of 0.053 (*104*). Major element and H_2_O contents (118 μg/g) for DMM were taken from Salters and Stracke (*81*), while DMM S content is taken to be 150 μg/g (*109*).

We calculated three different mixing models, which together explain much of the variability of mantle S and H_2_O content estimates (Fig. 6C). All models assume 15 wt % H_2_O in the slab component, which is reasonable for a H_2_O-saturated sediment melt at 700° to 800°C (*98*), while the S content is varied from 0.1 wt % (model 1) to 0.7 wt % (model 3) and 1.5 wt % (model 2). Slab component H_2_O/S ratios in our models are 150 (model 1), 21 (model 3), and 10 (model 2). The ferric iron ratio (Fe^3+^/ΣFe) of the sediment melt was also varied, from 1 (model 1) to 0.6 (models 2 and 3). We used variable fluid S^6+^/ΣS values between 0.66 (model 2) and 1 (models 1 and 3).

A high slab component H_2_O/S (model 1) ratio is necessary to explain low mantle source S contents coupled with high mantle H_2_O contents in some volcanic systems. Such a slab component is realistic if no or only a minimal amount of fluid phase is present alongside an oxidized slab melt containing relatively low concentrations of dissolved sulfate while carrying a large amount of dissolved H_2_O. This would restrict the S-carrying capacity of the slab component relative to a fluid-rich scenario, where large quantities of S may partition into the fluid phase because of the large fluid/melt partition coefficient of S (*110*). A lower H_2_O/S ratio (models 2 and 3) is needed at localities where the mantle wedge is enriched in S, including Colima, Shishaldin, Augustine, Makushin, and Volcano D from Tonga. At these localities, the presence of a fluid-saturated slab melt with a large fluid fraction could provide an effective pathway for S into the mantle wedge. We propose that much of the variability of mantle wedge S contents, and ultimately, the S content of primary arc magmas, is controlled by the fluid/melt ratio of the slab component.

It has been proposed that oxidized slab melts and fluids carry sulfate ions (*20*, *23*–*28*, *33*), which drive oxidation in the mantle and could result in a positive correlation between mantle S and Fe_2_O_3_ contents (Fig. 6D). This is due to sulfide being the stable S-bearing phase in mantle peridotites, unless the sulfur-sulfur oxide (SSO) buffer is crossed, as suggested by Mungall (*16*). Sulfate addition to the mantle below the SSO redox buffer would result in concurrent oxidation of Fe^2+^ ions to Fe^3+^ in silicates and sulfide formation. In our model, we assume that before any melting, the mantle wedge remains in the sulfide stability field, and therefore, any sulfate from the slab component is reduced to S^2−^ while oxidizing Fe^2+^ to Fe^3+^. Therefore, the addition of a S-rich slab component with high S^6+^/ΣS will rapidly increase mantle Fe_2_O_3_ contents, as each mole of S^6+^ will oxidize 8 moles of Fe^2+^. Excess oxidation by ferric iron may result in the mantle wedge passing the SSO buffer and leaving the sulfide stability field (*16*), upon which S^6+^ becomes stable in the mantle. In this scenario, we hypothesize that S^6+^ would be present in a low fraction melt phase. At this stage, the slab-derived S^6+^ loses its oxidative potential in the mantle wedge, in a similar way to carbonate upon reaching carbon-CO/CO_2_ buffer at an *f*o_2_ near FMQ (*16*). This process may have occurred in the source of our most oxidized localities (including Makushin, Shishaldin, Volcano A, and Volcano L), which all fall on the shallowest slope in Fig. 6D and cannot be reproduced by S-driven oxidation only, and hence requires Fe^3+^ addition to the mantle wedge. Some arc systems with high modeled mantle wedge S content, including Colima and Augustine, require that not all S in the slab component is present as S^6+^. Therefore, for model 2, we assumed S^6+^/ΣS values of 0.66, realistic for a moderately oxidizing slab component in equilibrium with AOC at an *f*o_2_ below FMQ+1 (*33*), which causes a steeper increase in mantle S contents relative to Fe_2_O_3_. While some arcs may require ferric iron as an additional oxidant, experimental data suggest that sediment and AOC melts are both Fe-poor [<1.5 wt % total FeO (*98*, *99*)], limiting ferric iron–driven oxidation in the mantle wedge (and, consequently, the formation of an oxidized but S-poor mantle source). Nonetheless, our mantle melting and two-component mixing models clearly indicate that S is a critical oxidant in most subduction zones and that at least two-thirds of the S added to the mantle wedge must be in the form of sulfate to reproduce observed trends.

### Origin of S in the mantle wedge revealed by S isotopes

Previous studies on S isotopes in arc magmas from the Cascades (*25*), Kyushu (*26*), and Central America (*27*) imply that elevated δ^34^S values in arc magmas relative to MORBs are a global feature and have a mantle origin. While correlations between slab tracers, redox, and δ^34^S exist on an interarc basis (*25*, *26*), they are less significant on a global scale (*27*). Because of the highly variable geochemistry of arc magmas (Fig. 3), subduction zone thermal regimes (*37*), and sedimentary inputs (*85*), a more comprehensive understanding of the origin of elevated δ^34^S in arc magmas can only be achieved through the systematic study of arc volcanic systems from various subduction zones that have a wide range of inputs, thermal characteristics, and geometries.

**Fig. 7.**
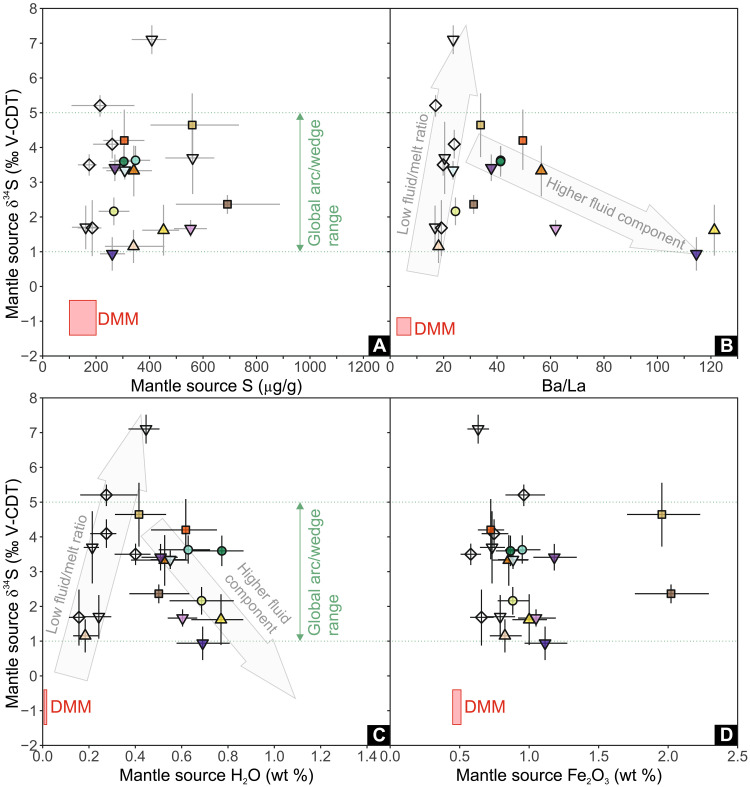
S isotopic composition of the subarc mantle. The estimated median S isotopic composition of the mantle source of various volcanic systems is shown against modeled mantle S content (**A**), slab tracer Ba/La (**B**), and modeled mantle H_2_O (**C**) and Fe_2_O_3_ contents (**D**). Because of total S loss from the mantle source, we assume that the δ^34^S value of primary arc melts, presented in Fig. 1, reflects that of the mantle wedge. Error bars are the same as defined in Fig. 5 for melting degrees: 70% confidence intervals taken from our Monte Carlo simulation. S isotope data for Lassen and Kyushu are from Muth and Wallace (*25*) and Kawaguchi *et al.* (*26*). The red rectangles represent the composition of the DMM, with values taken from Labidi *et al.* (*45*) for δ^34^S, Davis and Cottrell (*104*) for Fe_2_O_3_, Ding and Dasgupta (*109*) for S, and Salters and Stracke (*81*) for H_2_O and Ba/La. Green dotted lines indicate the global arc magma/mantle wedge δ^34^S range at +3 ± 2‰. Symbols are the same as in Fig. 6.

In addition to existing δ^34^S data from the Cascades, Central America, and Kyushu, we present δ^34^S data from three additional arcs: the Aleutians, where variable quantities of terrigenous and pelagic sediments subduct [on the basis of data from ODP886 and IODP U1417 drill sites (*111*, *112*)]; the Marianas, where an old, cold, and sediment-rich slab subducts [ODP 802A (*31*)]; and Tonga, which is a global end-member subduction zone with respect to both the thermal structure [coldest (*37*)] and sedimentary inputs [thinnest, on the basis of data from the IODP 1365 drill site (*113*)]. Our undegassed melt δ^34^S estimates from these three contrasting arcs overlap within uncertainty. Our data and previously published values together define the global δ^34^S range of arc magmas between +1 and +7‰, with most (17 of 20) within the +3 ± 2‰ range (Fig. 7). All arcs are enriched in ^34^S relative to MORB [−0.9‰ (*45*)], the composition of which is thought to represent the ambient upper mantle. Our data alongside literature values demonstrate that high δ^34^S values are a global feature of subduction-related magmas, irrespective of sediment input or other subduction zone parameters such as oceanic plate age and thermal structure. Crucially, our arc magma δ^34^S estimates can be taken as representative of the δ^34^S value of the mantle wedge; as in our models, we assume total S loss from the wedge because of melting. Therefore, any isotope fractionation during melting can be ruled out. We also consider the potential effect of sulfide retention in the mantle wedge after melting on S isotopes using equilibrium closed system isotope fractionation models (*17*) at 1300°C (fig. S11). A maximum difference of −2.5‰ between arc primary melt and initial mantle wedge δ^34^S is theoretically possible if most sulfide is retained in the source and the primary melt contains only S^6+^. However, this scenario is unrealistic because our high melting degree (Fig. 5) and primary melt S solubility estimates suggest that sulfide will be removed from the residue effectively and cannot be retained in large fractions. At a 40% residual sulfide fraction, the maximum isotope fractionation between melt and the mantle source is only −1‰, with a diminishing isotope fractionation as more sulfide is removed. At lower *f*o_2_ and melt S^6+^/ΣS, when sulfide retention in the source is more plausible, isotope fractionation is smaller—for example, at 50% residual sulfide fraction in the source and S^6+^/ΣS of 0.5, the melt δ^34^S is only 0.6‰ lower than the initial mantle wedge (fig. S11). Therefore, we argue that the presence of residual sulfide can only cause minor bias (underestimation) in our mantle wedge δ^34^S estimates.

Considering that the six arcs in this dataset include localities that carry both ^34^S-depleted sulfide-rich [bulk δ^34^S at −19.5‰ in Central America (*34*)] and ^34^S-enriched sulfate-rich sediments [bulk δ^34^S at +12.5‰ in the Marianas (*31*)], our narrow global arc δ^34^S range (+1 to +7‰) is notable. There is no linear relationship between trace element ratios, mantle wedge S, H_2_O, or ferric iron content, and δ^34^S (Fig. 7) on a global scale. However, a more complex relationship between H_2_O, Ba/La, and δ^34^S can be observed for groups 1 and 2, which we define on the basis of their trace element composition in Fig. 3. Group 1 volcanoes (high Ba/La and low Nb/Zr) have decreasing δ^34^S with increasing H_2_O and Ba/La, while group 2 localities have increasing δ^34^S values at higher H_2_O content and Ba/La. These correlations may indicate some difference in the S isotopic composition of melts and fluids originating from the slab, with fluid-poor oceanic crust melts (sampled by group 2) having a higher δ^34^S and H_2_O-rich slab fluids (sampled by group 1) having a comparatively lower δ^34^S (Fig. 7, B and C). This trend may be explained by more effective mobilization of slab S at localities with high H_2_O and Ba/La, resulting in less S being retained in the slab after devolatilization and smaller degrees of isotope fractionation (*28*), pushing the slab component’s δ^34^S value toward the slab’s bulk isotopic composition, which is in turn dominated by the AOC and unaltered oceanic crust (*24*, *34*).

**Fig. 8.**
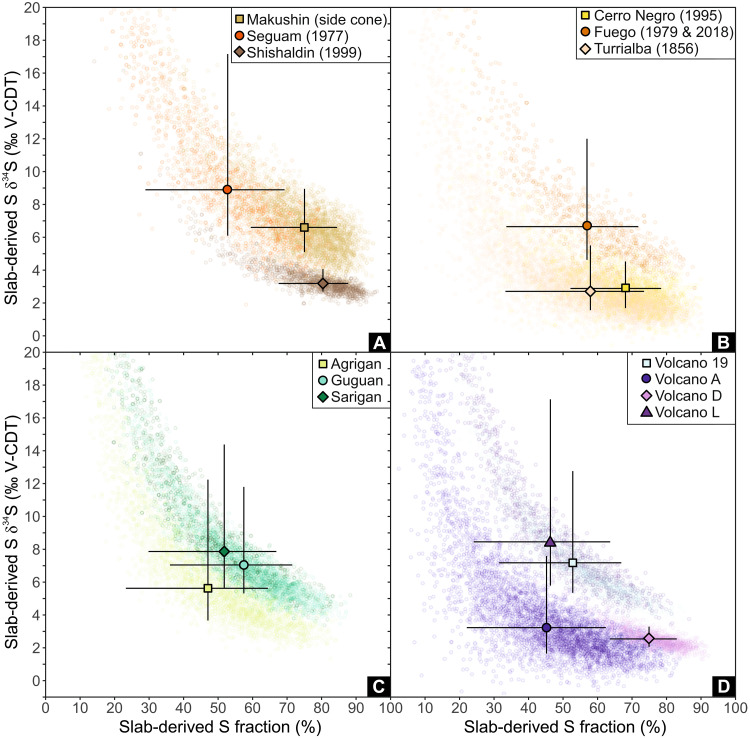
Modeled slab-derived S proportions in the mantle and the S isotopic composition of the slab component. Modeled slab component δ^34^S plotted against the percentage of slab-derived S. Values plotted were derived from Monte Carlo simulation–based mass balance modeling. Each plot shows data from one of the four studied arcs: Aleutian Islands (**A**), Central America (**B**), Mariana Islands (**C**), and Tonga (**D**). Smaller transparent symbols are individual model outcomes from our Monte Carlo simulation (*n* = 5000 for each locality), while larger symbols are the median values. Error bars are the same as defined in Figs. 6 and 7.

**Fig. 9.**
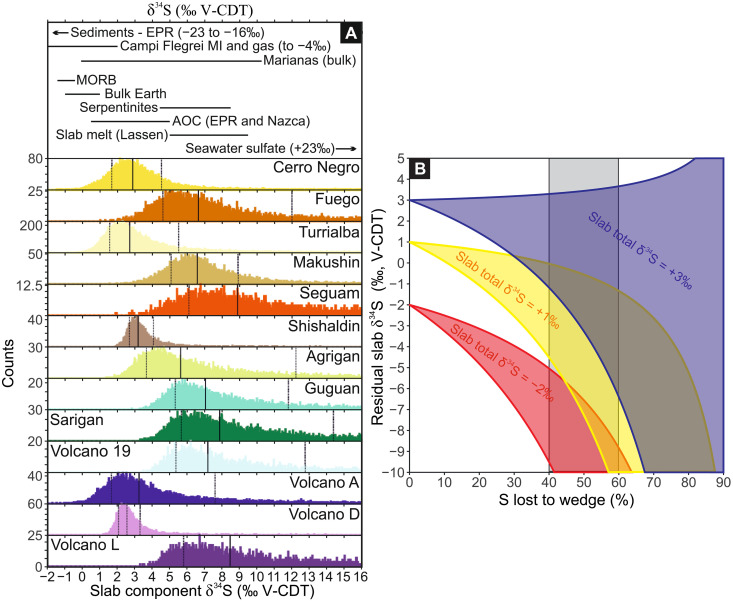
Estimates of slab component and residual slab δ^34^S values. In (**A**), each histogram shows the result of our Monte Carlo simulation for the slab component—for each primary melt composition, we calculate 5000 outcomes. At the top of the figure, δ^34^S intervals of various mantle reservoirs and surface lithologies are shown: sediments from the East Pacific Rise taken from Peccia *et al.* (*34*), volcanic gases and MIs from Campi Flegrei (*55*), MORB (*45*), bulk submarine and subaerial rocks from the Marianas (*47*), bulk Earth based on chondrites (*17*), serpentinites (*24*), AOC near Central America (*24*), slab melt estimate for the Lassen volcanic complex (*25*), and Cenozoic seawater sulfate (*136*). Solid black lines indicate the median slab component δ^34^S value, while dashed lines are 15 and 85% values of the distributions. In (**B**), the δ^34^S value of the residual slab is estimated as a function of S loss using the slab component δ^34^S range shown in (A): minimum of +2.6‰ at Volcano D and maximum of +9.2‰ at Seguam. Calculations are based on the same mass balance approach as for the slab component. To demonstrate the potential effect of S isotope heterogeneity in bulk slabs, we calculated models using three initial slab δ^34^S values: +1‰ [estimate of Peccia *et al.* (*34*) for the modern Central American arc; yellow field], +3‰ (blue field), and −2‰ (red field). The horizontal gray bar indicates 40 to 60% S loss from the slab on the basis of the estimate of Peccia *et al.* (*34*).

To estimate slab-derived S fractions and the δ^34^S values of slab components (Fig. 8) within the mantle wedge, we use the same slab component-DMM mixing model used to model mantle wedge Fe^3+^-H_2_O-S relationships (Fig. 6) using the same approach as in (*27*) for the Central American arc. We assume a DMM S content of 150 ± 50 μg/g (*109*) and a δ^34^S of −0.9‰ (*45*). These values are combined with our modeled mantle wedge S contents and δ^34^S values and then used to calculate the slab-derived S fraction and slab component δ^34^S in the mantle wedge. Here, we provide a simple example of this calculation: At Shishaldin (Virgin cone), we estimate the mantle wedge S content at 693 μg/g, which is 543 μg/g above the DMM estimate. This excess S is assigned to the slab component, which means that 80.7% of the total wedge S budget comes from a slab source. Shishaldin (Virgin cone) has a δ^34^S value of +2.4‰, which is 3.3‰ above the DMM estimate. Using mass balance, we can calculate that the 80.7% slab S fraction in the source must have a δ^34^S of +3.2‰ to produce a bulk mantle wedge with a δ^34^S value of +2.4‰. This calculation is carried out 5000 times for each locality, the results of which are presented in Figs. 8 and 9 for the Aleutian, Central American, Mariana, and Tonga arcs. In the case of residual sulfide being present in the source, our slab component δ^34^S values are maximum values, as a higher mantle wedge S content would require lower slab δ^34^S to explain the same mantle wedge δ^34^S value.

As previously suggested for Central America (*27*), we find that a considerable portion of S in the mantle wedge worldwide is slab-derived: Median values for each locality are between 27% (Fukue, Kyushu) and 86% (Colima, Mexico). From the 29 localities in our dataset, 18 have slab-derived S fractions above 50%, highlighting the importance, and in many cases, dominance, of slab-derived S in the mantle wedge and, consequently, in arc magmas. For simplicity, we use a single S content for the upper mantle end-member composition in our binary mixing model. This assumption may bias slab-derived S fraction estimates toward higher and lower values in localities that potentially contain elevated [because of the presence of pyroxenites (*92*)] or decreased [because of melting in the back arc (*40*)] S contents in their ambient mantle source. Little is known about the S content of recycled mantle components, such as eclogites and pyroxenites: On the basis of previous deep-mantle recycling efficiency estimates for S of 40 to 60% and using a bulk S content estimate for the subducting East Pacific Rise of 816 μg/g (*24*), a recycled slab in the deep mantle would contain 330 to 490 μg/g S, around two to three times as much as the DMM. Such recycled components could cause an overestimation of the slab S fraction for localities falling within group 3 (characterized by elevated Nb/Zr) in Fig. 3. For example, the addition of 10% recycled crust with 490 μg/g S into 90% DMM would raise the upper mantle S content to 184 μg/g. Melting in the back arc under reduced conditions would deplete the upper mantle in S. At Tonga, Cooper *et al.* (*40*) estimated between 4 and 5% melting in the back arc to form the ultradepleted mantle source of Ti-poor boninites, including Volcano A and Volcano L in our δ^34^S dataset. Assuming a silicate melt S solubility of 1000 μg/g during melting in the back arc, 4 to 5% melting will remove 40 to 50 μg/g S from the mantle source, resulting in a 33% decrease in mantle S contents entering the arc relative to DMM. At localities such as Volcano A and Volcano L, we may underestimate the slab-derived S fraction. Nonetheless, these estimates would still be within the uncertainty of the DMM estimate we use (150 ± 50 μg/g) in our Monte Carlo simulation and would not change any of our estimates within the error or influence our interpretation.

The S isotope ratio of the slab component must be elevated relative to the DMM value of −0.9‰ to satisfy undegassed arc magma δ^34^S values (Fig. 7). Using the results of the mixing model, we calculate the δ^34^S of the slab component for all studied localities (Fig. 8) and literature data available from the Cascades and Kyushu (*25*, *26*). The highest slab component δ^34^S values are for Lassen (Cascades), with median values across four eruption centers between +7 and +15‰. These δ^34^S values have high uncertainties (70% confidence intervals larger than 20‰) because of the low mantle wedge S content estimates for Lassen samples (Fig. 6), which all have large relative errors. We also estimate a high slab-derived δ^34^S value for the Aso caldera in Kyushu, between +10 and +16‰ (median at +11‰). For all other localities, slab-derived δ^34^S estimates fall within a comparatively narrow range: between +2‰ at Volcano D (Tonga) and +9‰ at Seguam (Aleutians) (Figs. 8 and 9). Overall, the δ^34^S of most arc volcanoes can be explained by the addition of a slab component with a δ^34^S of +5 ± 3‰. Errors associated with these estimates are demonstrated in both Figs. 8 and 9A: Uncertainties scale with estimates of the slab-derived S fraction with comparatively low errors for localities that are slab-dominated, such as Volcano D (Fig. 8D).

Residual slabs are expected to sink into the deeper mantle and consequently drive the S concentration and S isotope heterogeneity in the mantle source of ocean island basalts (*11*, *114**–**116*). Using our slab component δ^34^S estimates (Fig. 9A), we can calculate the δ^34^S of the residual slab as a function of S loss from the slab (Fig. 9B). Assuming that the initial δ^34^S of the slab is between −2 and +3‰ (see the caption of Fig. 9 for the justification of this range) and a 50 ± 10% S loss from the slab, we calculate that the bulk residual slab in modern subduction zones has a maximum δ^34^S of +4‰, and values below DMM estimates (<−1‰) are common (down to −10‰). Considering that much of the S budget of the slab is present in the AOC and primary oceanic crust (*24*, *34*), and the processes that form these lithologies have been similar through geological time, we hypothesize that slabs that entered ancient subduction zones and are the source of lithological heterogeneities currently sampled by intraplate magmas had bulk δ^34^S similar to modern slabs. Some ocean islands sample recycled components with comparatively negative δ^34^S (<−1‰), including Pitcairn Island (*116*) and Mangaia in the Cook Austral Islands (*115*), while other localities are expected to contain recycled components with δ^34^S values above the DMM estimate (>−1‰) such as El Hierro in the Canary Islands (*11*). Except for data from El Hierro, most ocean island S isotope analyses are derived from bulk rocks or sulfides. As we demonstrate, bulk samples are likely influenced by degassing, which may drive δ^34^S toward either negative or positive values as a function of melt redox (Fig. 2), while sulfides may also be affected by 1 to 2‰ isotope fractionation if the melt was somewhat oxidized and S-rich. Our results highlight that combining MI, sulfide (if present), and bulk δ^34^S data at any volcanic system is necessary to provide strong and accurate constraints on the origins of mantle S heterogeneity.

While there is more than 20‰ variability between the δ^34^S of sedimentary inputs at the Central American and Mariana arcs (*31*, *34*), there is substantial similarity between slab-derived δ^34^S values at these two arcs (estimates at each system are within uncertainty; Fig. 9), alongside the Aleutian and Tonga arcs (all are within ±3.2‰). These arcs not only cover a wide range of sedimentary inputs but also much of the global variability in slab age (Neogene to Late Jurassic), thermal parameter (10 to 140), and depth below arc [66 to 141 km (*37*, *117*)]. On the basis of our data, it is unlikely that a sedimentary source dominates the S budgets of volcanic arcs, as in this case, slab component δ^34^S values should vary strongly from one arc to another. This sets S apart from other fluid-mobile elements such as Ba and Th or C, for which subducting sediments are large contributors (*85*, *118*, *119*). Mass flux estimates from the East Pacific and Nazca plates suggest that around 5% of the total S budget of subducting slabs is stored in sediment, while together, the altered and primary igneous crust will contain ∼80% of the total subducting S budget (*24*, *34*). Unlike sediments, which are highly variable from one subduction setting to another, the S isotopic composition of the oceanic crust is expected to be less variable between different localities, as the same processes (seawater and hydrothermal alteration and microbially mediated sulfate reduction) govern its composition globally. However, neither the AOC nor the primary igneous crust contains sufficient sulfate or ^34^S (*24*, *34*, *35*, *120*) to supply arc magmas with a sulfate-dominated slab component enriched in ^34^S, as predicted by our model calculations (Figs. 6, 8, and 9). Direct addition of seawater or seawater-derived barite with δ^34^S near +23‰ is unlikely according to our model, as our slab component δ^34^S estimates (+5 ± 3‰) are substantially lower. This interpretation is supported by the high S content (*121*) and positive (>+13‰) δ^34^S values (*122*) observed in Mariana forearc mud seeps, indicating that a considerable amount of seawater-derived S originally present in the subducting slab is lost before it reaches the arc.

Thermodynamic models guided by laboratory experiments indicate that oxidizing slab fluids containing Ca and Na ions have a large sulfate-carrying capacity (*123*), and equilibration between these sulfate-rich fluids and residual pyrite may drive S isotope fractionation, resulting in an up to 10‰ increase in slab fluid δ^34^S values (*28*). The largest isotope fractionations are for small amounts of S removed from the slab—considering the high S-carrying capacity of oxidizing slab fluids, this scenario is most likely if the fluid/melt ratio in the slab component is low. If the primary igneous crust retains its MORB-like isotopic composition near −1‰, a fractionation of +3 to +10‰ is necessary to produce slab components with high δ^34^S to supply arcs, while using an AOC δ^34^S value of +3‰, estimated in (*24*) for the East Pacific Rise, a smaller fractionation of 0 to +6‰ is sufficient. Smaller δ^34^S fractionation is favored for source lithologies with similar δ^34^S to the slab component, for high temperatures, and for a large degree of S mobilization from the slab (*28*), with only a small fraction of S returning into the deep mantle; such a scenario should become more likely if the slab component has a high fluid/melt ratio and S-carrying capacity. The latter interpretation is supported by our data, indicated by the decrease in slab component δ^34^S estimates for volcanoes with high Ba/La and mantle H_2_O content estimates (Fig. 7, B and C). In case of negligible S isotope fractionation, the material that enters the deep mantle should retain δ^34^S values similar to its initial value, albeit containing less S. On the other hand, if the source lithology of the slab components has a lower δ^34^S value, similar to primary igneous crust or to microbially mediated sulfides in the AOC (*35*), then stronger S isotope fractionation is necessary during the formation of the slab component. This scenario would favor colder conditions and the loss of a lower fraction of S from the original subducting lithology (*28*), resulting in residual material with low δ^34^S values reaching the deeper mantle. On the basis of our δ^34^S measurements and model calculations, both scenarios are realistic and feasible in subduction settings today. The above outlined processes are also in line with recent estimates of deep-mantle, recycled AOC component δ^34^S values near +2‰ in the mantle source of Canary Islands magmas (*11*), which could be generated by a small degree of S isotope fractionation in AOC with a +3‰ starting δ^34^S during subduction. Our S isotope data from arc magmas may be explained by either a low or high degree of S loss from the slab. Constraining the efficiency of S recycling into the mantle wedge at subduction zones requires further work with a focus on the S and S isotope mass balance of subduction zone inputs and outputs globally, such as work done on Central America (*24*, *34*). Nonetheless, our results provide crucial evidence of a globally prevalent process: the removal and oxidation of S from the subducting oceanic crust as the cause of S enrichment and the oxidative nature of arc magmas. This process is among the main pathways through which Earth retains an oxidized surface enriched in ^34^S over geological timescales. At the same time, the subduction zone S cycle likely limits the recycling of sulfate and Fe^3+^ into the deeper mantle (*124*, *125*). Therefore, the subduction zone S cycle effectively returns oxidized materials to the surface, rather than subducting them into the deeper mantle where they can be sequestered for geological time.

The role of S cycling in modulating redox processes on Earth first became important in the Archean with the emergence of anoxygenic photosynthesizing ancient cyanobacteria. These organisms produced oxidized species of Fe (Fe^3+^) and S (elemental S^0^) on the surface, followed by oxygenic photosynthesis at a later stage (*126*). Before the Great Oxidation Event 2.5 billion years ago, all biogenic oxygen is expected to have been consumed by Fe^2+^ dissolved in the early oceans, depositing Fe^3+^ on the seafloor. This process is recorded by Neoarchean banded iron formations that formed on continental shelves (*127*), but Fe^3+^ deposition would also have occurred in the deep sea at the same time. This process, combined with the emergence of plate tectonics, may have resulted in the first oxidized materials being transported from the surface to the mantle. As our results suggest for modern subduction, reactions between Fe^3+^ and S in the slab during melting and devolatilization, followed by sulfate addition to the mantle wedge, melting, and lastly, magma degassing, would have returned oxidized S to the surface, aiding atmospheric oxygenation during this critical period in Earth’s history. In this scenario, the subduction zone S and redox cycles are linked directly to the evolution of the early biosphere. Furthermore, because the subduction zone S cycle not only returns oxidized material to the surface but is also expected to subduct considerable amounts of reduced S into the mantle (Fig. 9B), it restricts the amount of S present on Earth’s surface. This contrasts with other planetary bodies in our Solar System like Venus, which has active volcanism but lacks plate tectonics and biological activity. At Venus, it is possible that constant degassing of S from volcanoes has resulted in S accumulation on the surface and in the atmosphere. The lack of a return mechanism (subduction) or a process that can fix S as sulfide in the crust (biological activity) means that there are no negative feedbacks countering this S accumulation. This is in contrast to the subduction- and biosphere-modulated S cycle on Earth.

### S in arc magmas and the mantle wedge

Our analyses and model calculations demonstrate that subducting slabs contribute large quantities of S to the mantle wedge and, consequently, to arc magmas and surface environments via volcanism. Slab-derived S can elevate the mantle’s S content from 100 to 200 μg/g to above 900 μg/g in extreme cases (Fig. 6). Using our S isotope data in conjunction with degassing models, we demonstrate that a systematic relationship exists between magma redox and the S isotopic evolution of volcanic gases (Figs. 1 and 2). The effects of isotope fractionation must therefore be considered during the interpretation of S isotope data collected from volcanic gases. Using S isotopes, we also predict the tipping point at which S degassing is no longer able to reduce silicate melts, which is close to a melt Fe^3+^/ΣFe ratio of 0.25 (>FMQ+2). We define the global δ^34^S range of arc magma and the mantle wedge at +3 ± 2‰, which is enriched in ^34^S relative to the upper mantle [−0.9‰ (*45*)]. Combining trace element ratios, arc primary melt, and mantle wedge S, H_2_O, and ferric iron content estimates, we show that S is added to the mantle wedge mostly in oxidized form (as sulfate; Fig. 6). Alongside our S isotope data, these results support thermodynamic model results (*28*, *33*) suggesting that oxidizing fluids formed within the subducting oceanic crust are critical transporting agents of downgoing slab-derived S. We hypothesize that the degree of ^34^S-enrichment in arc magmas relates to the melt/fluid ratio of the slab component: Lower δ^34^S values may be characteristic of localities where a fluid-rich slab component effectively removes S from the slab, limiting isotope fractionation between the slab component and the solid residue. Variable degrees of S removal from the slab would result in compositionally diverse residual slabs entering the deep mantle. The estimated δ^34^S of slab components falls mostly in the +5 ± 3‰ range globally (Fig. 7), which is unexpectedly narrow considering the variable S isotopic composition of sedimentary inputs [−20 to +13‰ (*31*, *34*)] in the studied arcs. While our models do not provide quantitative subduction fluxes of S, ferric iron, and H_2_O, and we make certain assumptions regarding the S isotopic composition of subduction zone input (particularly the AOC) and the melting process in the mantle wedge, our results provide a globally robust model of the subduction zone S cycle. Our results regarding (i) the global prevalence of the removal and oxidation of S from the subducting oceanic crust as the cause of S enrichment and the oxidative nature of arc magmas and (ii) the subduction zone S cycle likely limiting the recycling of sulfate and Fe^3+^ into the deeper mantle highlight how the subduction S cycle has helped to maintain Earth’s oxidized surface over geological time by effectively returning oxidized materials to the surface via slab-mantle interaction, mantle melting, and volcanic degassing.

## MATERIALS AND METHODS

For this study, we used samples collected from four volcanic arcs, representing 13 volcanic systems. All these samples were used and characterized in previous studies (*27*, *40*, *42*, *63*, *128*–*132*). We used four samples from three volcanoes from the Aleutian Islands: Pakushin side cone, Makushin volcano, Seguam (1977 eruption), Shishaldin (1999 eruption), and Virgin Side cone, also from Shishaldin (*42*, *128*, *129*). MIs and whole-rock samples from the 1977 Seguam eruption were first characterized by Zimmer *et al.* (*4*), while the Makushin and Shishaldin samples are described by Rasmussen (*129*). The chemical composition of MIs from these samples is available as an EarthChem dataset (*42*). Mariana samples used in this work were collected as part of the National Science Foundation (NSF) Margins project from Agrigan, Guguan, and Sarigan. These samples have been previously studied for their volatile content (*94*) and iron speciation (*63*); whole-rock data from these samples are published as an EarthChem dataset (*130*). Details on the Tonga samples used in this study are provided by Cooper *et al.* (*40*), including MI volatile contents and trace element data. Samples used from Central America are described by Barth *et al.* (*132*) for Cerro Negro and Taracsák *et al.* (*27*). S isotope data collected from these samples were first published by Taracsák *et al.* (*27*).

Major element composition of the studied MIs (including S and Cl) and their host crystals from the Aleutian, Mariana, and Tonga arcs was determined using a CAMECA SX-Five field emission gun electron microprobe analyzer at the Department of Earth Sciences, University of Oxford. Analytical conditions for the EPMAs are detailed by Taracsák *et al.* (*27*). All data are provided in the Supplementary Materials, including analytical uncertainties.

Trace elements Li and B, alongside volatile contents (H, C, F, Cl, and S), were analyzed at the NERC Edinburgh Ion microprobe facility (EIMF) using an IMS-1270 and an IMS-7fGeo secondary ion mass spectrometer. Analytical conditions and calibration procedures were identical to those detailed by Taracsák *et al.* (*27*) for data collected in 2022 (mainly consisting of Aleutian glass analyses). In summary, these included analyses of F, Cl, and S as negative secondary ions using the IMS-1270 and a Cs^+^ primary beam. Li, B, H, and C contents were analyzed as positive secondary ions using the IMS-7fGeo instrument and a O^−^ primary beam.

Trace and volatile element contents collected in May 2024 (mostly Mariana and Tonga samples) were all determined using the IMS-7fGeo instrument at the EIMF. These analyses included a separate setup to measure C before other trace elements. To analyze C, we used a primary beam (O^−^) current of ∼5 nA with a 13-kV accelerating voltage. Secondary ions of ^24^Mg^2+^, ^12^C^+^, ^40^Ca^2+^, and ^30^Si^+^ were analyzed for 5, 10, 2, and 2 s, respectively, each for 15 cycles. Before analyses, the sample surface was presputtered for 240 s. Secondary ions were accelerated using 5 kV minus a 50-V sample voltage offset, which was used to eliminate any low-energy secondary ions entering the mass spectrometer. Trace and volatile elements (H, Li, B, F, P, and Cl) were analyzed using a O^−^ primary beam with a 5-nA current. Each ion was analyzed for 5 s for 10 cycles apart from F, which was analyzed for 10 s. Alongside the trace and volatile elements, ^30^Si^+^, ^44^Ca^+^, and ^26^Mg^+^ were also measured. The background for H^+^ was between 130 and 220 cps, equal to ∼0.03 wt % H_2_O, while the ^12^C^+^ background was 0.24 to 0.3 cps, equal to 9 to 11 μg/g CO_2_.

S isotope ratios in the glasses were measured in two laboratories between 2022 and 2024. Most Aleutian samples were analyzed at EIMF using an IMS-1270 instrument. Details of these analyses, including standard analyses, are detailed by Taracsák *et al.* (*27*). In summary, these analyses were carried out using a 3- to 4-nA Cs^+^ primary beam at 4800 MRP (mass resolving power). Ions of ^32^S^−^ and ^34^S^−^ were collected simultaneously using a faraday cup-electron multiplier detector setup.

The 2024 S isotope analytical session was carried out at the Northeastern National Ion Microprobe Facility, Woods Hole Oceanographic Institute. Detailed analytical protocols were identical to those described by Taracsák *et al.* (*11*). In summary, analyses were carried out on an IMS-1280 instrument in multicollection mode using a ∼200-pA primary beam, collecting ^32^S^−^ and ^34^S^−^ simultaneously on two electron multipliers. All of our Mariana and Tonga arc δ^34^S data were collected during this session, while a small subset of Central American (Fuego) and Aleutian (Shishaldin, Makushin) glasses was also analyzed to verify reproducibility between the two laboratories (fig. S7). We find that unlike by Taracsák *et al.* (*11*), data collected from the same samples in the two laboratories overlap. This is potentially due to the better calibration approach used in this study, including the analyses of standards with both high and low S contents in every session. Nonetheless, we excluded any data point collected at EIMF for which S content was below 500 μg/g, including those published by Taracsák *et al.* (*27*), as the errors associated with logarithmic, S content–dependent instrumental mass fractionation correction applied to these data are highly uncertain. Data points with low S contents have a large leverage on the shape of the regressions used to model degassing (Fig. 1), and their large errors caused by low count rates and data correction procedures would introduce further uncertainty into modeling S isotope fractionation during degassing. Further details on analytical procedures alongside a comparison of data collected in the two laboratories are provided in Supplementary Text.

## Supplementary Materials

### The PDF file includes:

Supplementary Text Figs. S1 to S12 Table S1 Legend for file S1 References
