# Supplementary material for: The sulfur content and isotopic composition of the subarc mantle
Source: Sci Adv. 2026 Jul 31;12(31):eaeb9747. doi: 10.1126/sciadv.aeb9747 (PMC13426414; doi:10.1126/sciadv.aeb9747)
Supplement: Supplementary file 1 — Supplementary Text Figs. S1 to S12 Table S1 Legend for file S1 References [file sciadv.aeb9747_sm.pdf]

Science Advances
AAAS

Supplementary Materials for

The sulfur content and isotopic composition of the subarc mantle

Zoltán Taracsák *et al.*

Corresponding author: Zoltán Taracsák, zt265@cam.ac.uk

Sci. Adv. **12**, eaeb9747 (2026)
DOI: 10.1126/sciadv.aeb9747

The PDF file includes:

Supplementary Text
Figs. S1 to S12
Table S1
Legend for file S1
References

Other Supplementary Material for this manuscript includes the following:

File S1

Supplementary Text

Melting degree estimation – further details. To investigate the S content of the subarc mantle, it is necessary to invert melt inclusion (MI) S contents to those of the mantle wedge. This requires the knowledge of melting degrees and the partitioning behavior of sulfur during melting.

We calculate melting degrees for the volcanic systems from which we analyzed $\delta^{34}S$ values, in addition to localities with literature data on major, trace, and volatile (S, H_2O) contents, as well as independent estimates of oxygen fugacity (fO_2). We utilize two independent approaches to calculate melting degree. One method uses non-modal batch melting and primary arc magma Na contents (Kelley et al (*137*) uses Ti in a similar approach), while our second approach relies on estimates of the last equilibration pressure and temperature (*78*) of primitive arc magmas with mantle olivine and a P-dependent parameterization of melt productivity (dF/dT) – detailed description of this approach are provided by Kelley et al. (*94*) and Cooper et al. (*40*).

As a first step, we calculate the primary melt compositions for each studied locality (102 melt compositions in total, see data sources in supplementary spreadsheet S3) via the addition of equilibrium olivine using the method of Lee et al. (*78*). This involves recalculation of primary arc magma compositions in equilibrium with olivine containing 91 (most localities) to 93 (boninites at Volcano A and L from Tonga) mol% forsterite. For these calculations use melt compositions (whole-rock and rarely, MI) that have >5.3 wt% MgO content to avoid using data that fall off the olivine-only liquid line of descent. Samples used to estimate primary melt compositions do not always represent the same eruption as the MIs that were analyzed for their H_2O and S contents. This is due to our data selection approach: where possible, we avoid using MI data to estimate primary melt compositions, as their Fe and Mg contents are often changed by diffusion (*138*), which makes temperature estimate from MIs unreliable. Calculated primary melt compositions are then used to estimate the P-T conditions of the final equilibrium between mantle olivine and primitive melt using the thermobarometer of Lee et al. (*78*) on a H_2O-free basis. In our Monte Carlo simulations, we use the errors quoted by Lee et al. (*78*) to quantify uncertainty of our melting degree estimates: ±3% relative error for temperature (±39 ˚C at 1300 ˚C) and ±0.1 GPa for pressure. Errors for melt fractions are between ±0.03 and ±0.07 (70% of the total distribution, Figure S9A).

The Lee et al. (*78*) model requires Fe speciation of the melt as an input. We estimate Fe speciation for each locality separately. If measured $Fe^{3+}/\sum Fe$ values from glasses are available (e.g. Marianas, *63*), we use these directly. We use uncorrected $Fe^{3+}/\sum Fe$ values (i.e. without PEC or Fe-diffusion correction), as different models and approaches are used to make these corrections in various publications – this ensures better comparability. If only direct fO_2 estimates are available (based on olivine-melt V partitioning or olivine-spinel oxybarometry), we convert an average fO_2 for each location to an $Fe^{3+}/\sum Fe$ value by using the equation of O'Neill et al. (*60*). We use the average whole-rock composition as an input melt composition for the conversion, while temperature needed to calculate log(fO_2) for the FMQ buffer is estimated using olivine-MI pairs from each locality. If only sulfur speciation data are available from a locality, we use the model of O'Neill et al. (*14*) to convert this to an $Fe^{3+}/\sum Fe$ value, again using the average whole-rock composition and olivine-MI based T as inputs.

As a final step, the "dry" T and P estimates are converted to "wet" estimates by applying a P-dependent correction for the cryoscopic effect of H_2O on mantle melting, calculated using primary melt H_2O contents (*2*). A dry solidus T is estimated using the method of Katz et al. (*87*), which is also offset by the same cryoscopic correction. We calculated F from the difference

between the estimated melt temperature and the wet-solidus temperature alongside a P-dependent dT/dF values taken from Cooper et al. (*40*).

To calculate melting degrees using primary melt Na contents, the partitioning behavior (D, P) and the concentration of Na in the primary melt and the source must be known. To calculate bulk D and P values for our non-modal batch melting model, we assume single mineral-melt partitioning coefficients values for olivine (0.0001) and spinel (0.0001). For clinopyroxene we used the P-dependent parameterization of Blundy et al. (*139*) to calculate Na partitioning coefficients, while for orthopyroxene we assumed the partitioning coefficients is 0.2 times that of clinopyroxene (i.e. a constant K_d between ortho- and clinopyroxene, *104*). We assumed a spinel peridotite lithology (0.54 olivine, 0.135 opx, 0.27 cpx, 0.055 spinel) with a melting reaction of -0.07 olivine, 0.3 opx, 0.72 cpx and 0.05 spinel. Based on the Dy/Yb ratios (between 1-2.5, similar to MORBs) of the studied systems and the last equilibration pressure estimates (<3 GPa) calculated using the thermobarometer of Lee et al. (*78*), we expect that all the studied systems last equilibrated with mantle olivine in the spinel stability field. The only exception for this is Colima, that has high Dy/Yb (>3) accompanied by moderate (~1.5 GPa) equilibration pressure. For pyroxene D values we assumed a 20% relative error in our Monte Carlo model calculations. We use variable mantle source Na contents: 1221 µg/g (0.165 wt% Na_2O) for depleted boninites at Volcano A and L from Tonga (based on 4.7% melt loss from DMM in the back arc, *40*), 2590 µg/g (0.349 wt% Na_2O, primitive mantle estimate, *135*) for group 3 localities that show OIB affinity (see main text) and 2152 µg/g (0.29 wt% Na_2O) for every other locality (DMM estimate, *81*). We assumed a 10% relative error for all three mantle source Na contents. For individual localities, melt fraction errors vary between ±0.014 and ±0.03 (70% of the total distribution, Figure S9).

Once both melt fraction estimates are calculated (the two methods are generally within uncertainty, Figure S9), the two melting degree estimates are averaged as part of the Monte Carlo simulations to derive a combined melt fraction estimate for each locality, which we present in the main text.

Using these melting degree estimates, we calculate mantle sulfur contents assuming perfect incompatible behavior during melting. At high fO_2, rapid consumption of sulfide from the mantle is expected due to the high total S solubility of melts – using our primitive melt compositions and estimates of melting T, we calculate that $S^{6+}/\Sigma S$ varies in primitive arc magmas from 0.3-0.4 at Sumiyoshi-ike to near 1 at most arc systems included in our dataset. Even at Sumiyoshi-ike, total S solubility of the melt under mantle conditions is estimated at >4000 µg/g, while our primitive melt S content estimate is below 3000 µg/g once olivine crystallization is accounted for. All other localities have higher $S^{6+}/\Sigma S$ and total S solubility is expected to be close to sulfur content at anhydrite saturation (SCAS), which is generally above 1 wt% S content for mafic magmas (*13,108*). Alongside S contents, we estimated the H_2O and Fe_2O_3 content of the mantle wedge, using non-modal batch melting. For H_2O, we used bulk D and P values of 0.012 and 0.019. These values were derived from mineral/melt partition coefficients published by Rosenthal et al. (*103*): their partition coefficients were averaged, resulting in mineral-melt partition coefficient values of 0.01438 for opx and 0.02097 for cpx. We use their single D value for olivine (0.0084), while for spinel we assumed a partition coefficient of 0.001, as we did not find any available literature data (nonetheless, H_2O is expected to be highly incompatible in oxides). Partition coefficients for ferric iron were taken from Mallmann and O'Neill (*140*) for olivine (0.0626), and from Davis and Cottrell (*104*) for cpx (0.78), opx (0.63), and spinel (using their equation 2). To calculate mantle H_2O and Fe_2O_3 contents we used the same mineral and melting proportions for phases as for the Na-based melting degree model calculations.

Redox is one of the most important parameters in our models. We demonstrate the relationship between fO_2 and $Fe^{3+}/\sum Fe$ in Figure S10B. Many of our fO_2 values presented in the main text are higher than those suggested for arc magmas in previous publications. For example, for the Los Hornitos cone in Quizapu, Tassara et al. (*77*) estimated fO_2 at FMQ+2.6 using Fe speciation data, while our estimate is FMQ+3.2. One of the reasons for this difference is that we use different conversion methods between $Fe^{3+}/\sum Fe$ and fO_2. Using the equation of Kress and Carmichael (*61*), we estimate $Fe^{3+}/\sum Fe$ values that are 0.02 to 0.07 higher than when we use the equation of O'Neill et al. (*60*) for the same melt composition (Figure S10B). These differences feed into our models, as $Fe^{3+}/\sum Fe$ is needed to estimate $S^{6+}/\sum S$ and total melt S solubility (main text, Figure 4). For localities where $Fe^{3+}/\sum Fe$ data is available, $S^{6+}/\sum S$ can be estimated directly and fO_2 conversion causes no further uncertainty in such cases. Where only fO_2 is available (i.e. based on olivine-melt V partitioning), using the equation of Kress and Carmichael (*61*) instead of O'Neill et al. (*60*) would result in higher $Fe^{3+}/\sum Fe$ estimates and in turn higher $S^{6+}/\sum S$, making sulfide saturation during melt evolution and sulfide retention in the source less likely for the studied arc magmas.

Sulfur isotope analysis and data processing. Sulfur isotopes data presented in this study were analyzed using large geometry secondary ion mass spectrometry (SIMS) at two laboratories: the NERC Ion Microprobe Facility, University of Edinburgh (2022) and Northeastern National Ion Microprobe Facility, Woods Hole Oceanographic Institute (2024). Most of our Aleutian glasses were analyzed at EIMF in 2022, while all Tonga and Mariana glasses, and a subset of Central American and Aleutian glasses were measured at WHOI in 2024. Detailed analytical procedures and rationale behind instrumental mass fractionation corrections for analyses carried out in 2022 are provided in the supplementary material of Taracsák et al. (*27*).

In summary, analyses in 2022 at EIMF were carried out using an IMS-1270 instrument operated in multi-collector mode, utilizing a faraday cup-electron multiplier detector setup to measure ^{32}S and ^{34}S ions. We used a 3-4 nA Cs^+ primary beam for the analyses. A regression between measured instrumental mass fractionation values (expressed as permil) and the ln(S) of each measured glass standard was fitted to estimate the S content dependent IMF observed using this setup (*27,141*). Raw SIMS sulfur isotope data are provided in the supplementary spreadsheets.

At WHOI, sulfur isotope analyses were carried out using an IMS-1280 instrument, operated in multi-collection mode with a Cs^+ primary beam and a current of 200-400 pA. Sulfur ions were collected using two electron multipliers alongside ^{30}Si ions. Based on analyses of at least three different standards with variable S content in each session, we determined that the sulfur-content dependent IMF was not present for these analyses, and therefore measured sulfur isotope ratios were corrected for only the constant offset observed between measured and bulk values from the standards. Further details on the WHOI SIMS analyses, including how S contents were quantified for SIMS analyses are provided in the methods section of Caliro et al. (*55*). Raw SIMS sulfur isotope data are provided in the supplementary spreadsheets.

To check the effectiveness ln(S)-based IMF correction applied to our $\delta^{34}S$ data collected in 2022, we have reanalyzed nine Aleutian melt inclusions at WHOI in 2024 (Figure S8). We find that there is a good agreement between EIMF and WHOI data for these MIs – most are within ±1‰, and all data fall on a 1:1 line within 1σ uncertainty. These observations contrast with that presented in Taracsák et al. (*11*) for Canary Islands glasses, that showed a significant scatter for glasses from which data collected at WHOI and EIMF were both available. A main difference in our study is that in all cases, we have analyzed at least one sulfur-rich (EGT17-01 at ~2900 µg/g

S content) and an S-poor (STAP at 500 µg/g) standards in each session at EIMF. We argue that by using both low and high S content standards in the calibration for each individual session, we were able to more accurately characterize the composition dependent IMF at EIMF than Taracsák et al. (*11*), who did not analyze S-poor standards in every session. Based on the overlap between corrected EIMF data and those measured at WHOI, we find no reason to exclude Aleutian and Central American glass data collected at EIMF in 2022 from our dataset. Our standard set analyzed at WHOI and Edinburgh included both natural Icelandic glasses with $\delta^{34}S$ values at -1‰ and synthetic glasses with reaching a $\delta^{34}S$ of +10‰ – this range covers our unknown dataset (from -1 to +11‰).

<u>Monte Carlo modelling of mantle melting and mantle-slab interaction.</u> In the main text we provide results of our melting and slab-mantle mixing model. Uncertainties for these models were carried out using Monte Carlo simulations in R. For each primitive arc magma composition (n = 102), we ran our melting degree model calculations for 5000 times, resulting in 510000 total outcomes. For the thermobarometer-based melting degree estimates, pressure and temperature estimates were varied, assuming Gaussian distributions and ±3% relative error for temperature and 0.1 GPa for pressure. For the Na based estimates, we varied D values for opx and cpx (20% relative error). Similarly, to estimate mantle source Fe_2O_3 contents, we used variable D values (assuming lognormal distribution for cpx, opx, and olivine to avoid negative values) in our Monte Carlo model. Primary arc melt S and H_2O contents were varied by 5% (relative), while Fe_2O_3 contents were varied by 10%, assuming Gaussian distribution (as for these concentrations it was unlikely that negative values are generated). For the depleted mantle (DMM) S content, we assumed a S content of 150 µg/g, a ±50 µg/g error and a lognormal distribution, while for $\delta^{34}S$ we assumed a ±0.5‰ error. For the slab components S content, we assumed a values of 5000±1000 µg/g (lognormal); we note that changing this value only changes the mass fraction of the slab component in the model calculations and does not significantly affect the slab-derived sulfur fraction or slab component $\delta^{34}S$ estimates. For all other values in the model, a single value was used (i.e. repeated 5000 times for each composition).

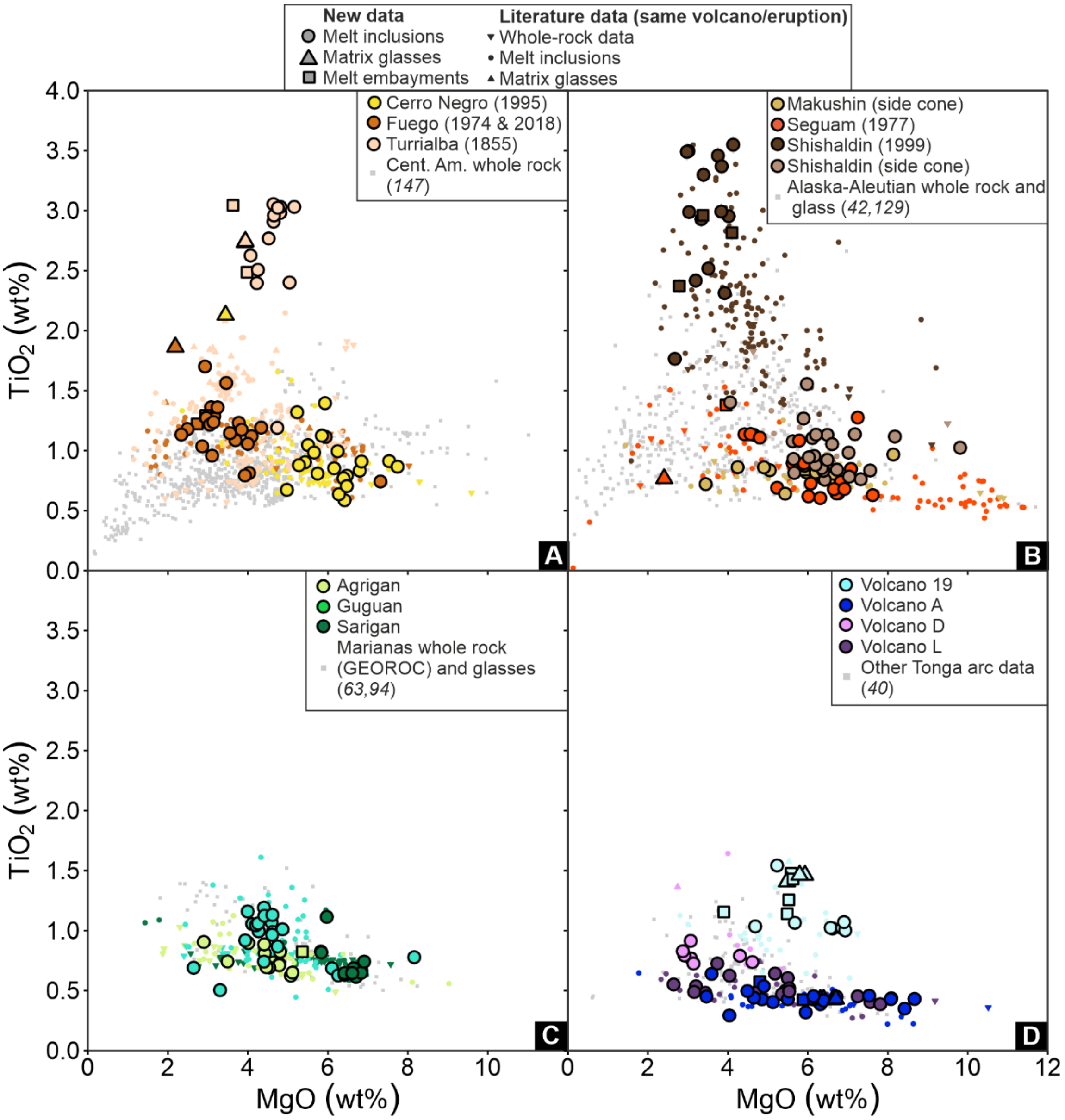

Figure S1: The composition of analyzed glasses on TiO_2 (wt%) vs. MgO (wt%) diagrams. Melt inclusion data were not corrected for post-entrapment processes. Each plot corresponds to data from a single arc (Central America – A; Aleutians - B; Marianas – C; Tonga – D). Most glass data in (A) were first presented in Taracsák et al. (*27*), with a smaller subset of new analyses. We also present previously published data for Central American locations (*128,132,142-146*) using smaller symbols, including melt inclusions (circles), matrix glasses (upwards triangles), and whole-rock samples (downwards triangles). Grey squares in (A) are data from other Central American volcanoes, taken from Carr et al. (*147*). In (B) we plot published data for the Aleutians (*4,42,129*). Grey squares in (B) are whole-rock data collated by Rasmussen (*129*). In (C) we also present published data for the Marianas (*63,94,148-154*) – grey symbols are data from systems that are not represented in our primitive arc dataset. Literature data for the Tonga (D) arc (including other volcanoes – grey squares) are from Cooper et al. (*40*). Errors are 1σ, and for most data, smaller than symbol size.

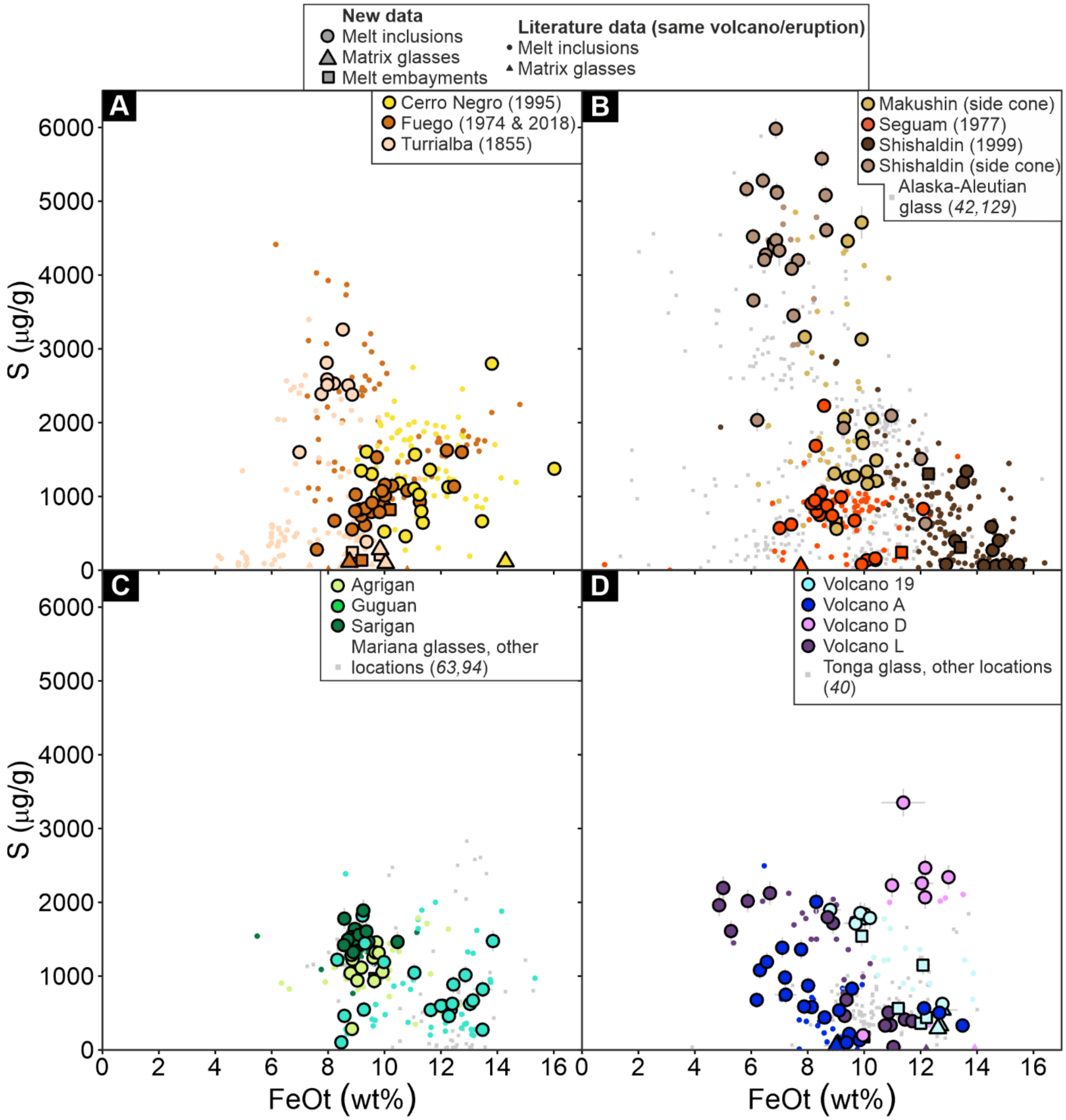

Figure S2: The composition of analyzed glasses on S (µg/g) vs. total FeO (wt%) diagrams. Data from Central America (A), Aleutians (B), Marianas (C), and Tonga (D) are provided in separate plots. No corrections were applied to the melt inclusion data presented here, as corrections may overwrite FeO-S relationship that could be indicative of pre- and post-entrapment sulfide saturation. Symbols and references for literature data are the same as described in the caption of Figure S1. Small colored symbols are literature data on glasses and melt inclusions from the same systems studied for $\delta^{34}S$, grey squares show glass/MI data from the same arc but other volcanoes.

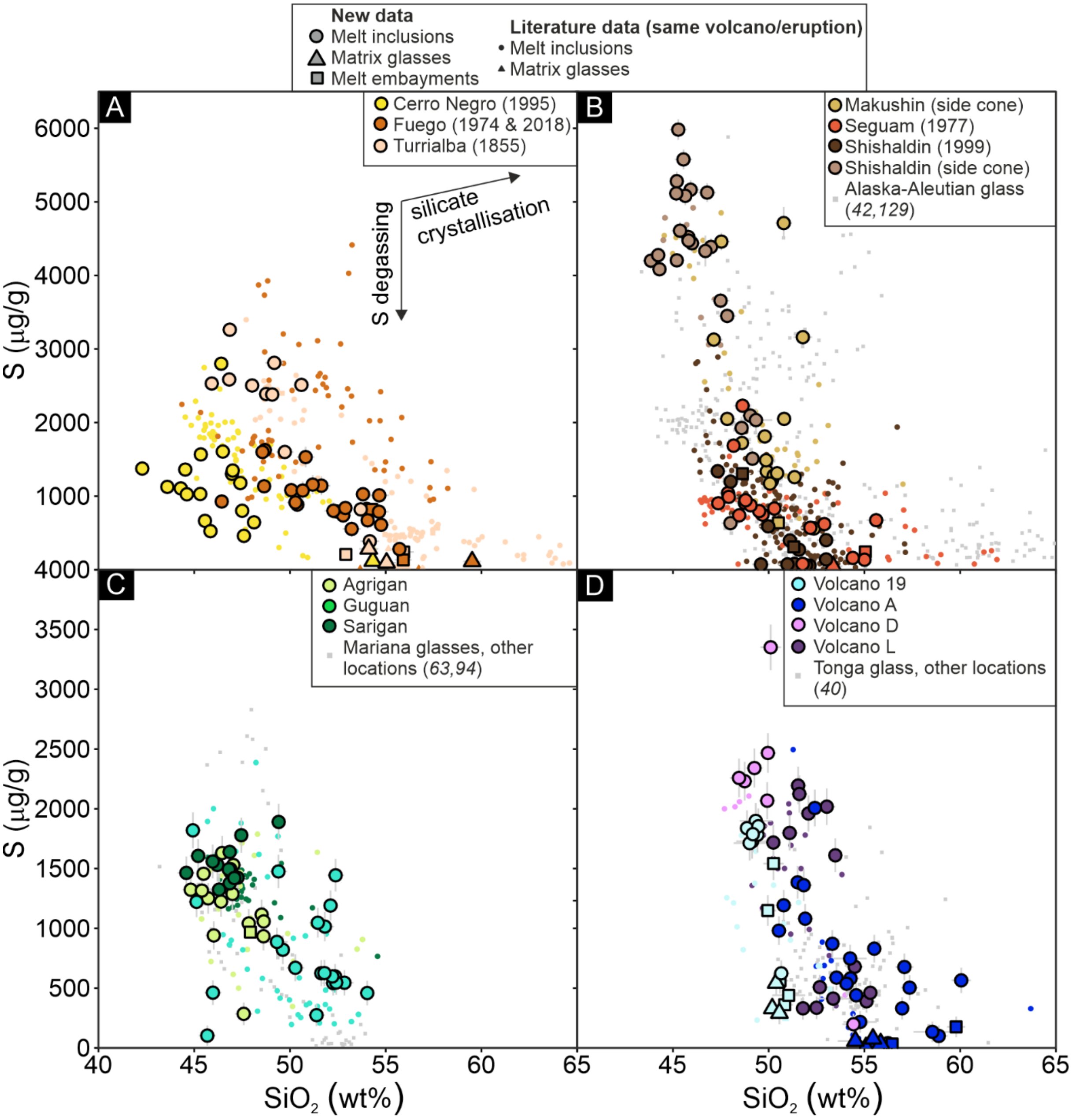

Figure S3: The composition of analyzed glasses on S (µg/g) vs. SiO_2 (wt%) diagrams. Data from Central America (A), Aleutians (B), Marianas (C), and Tonga (D) are provided in separate plots. Arrows in (A) show approximate vectors of crystallization (concurrently increases S and SiO_2 contents) and degassing (decreases S at constant SiO_2). Symbols and references for literature data are the same as described in the caption of Figure S1.

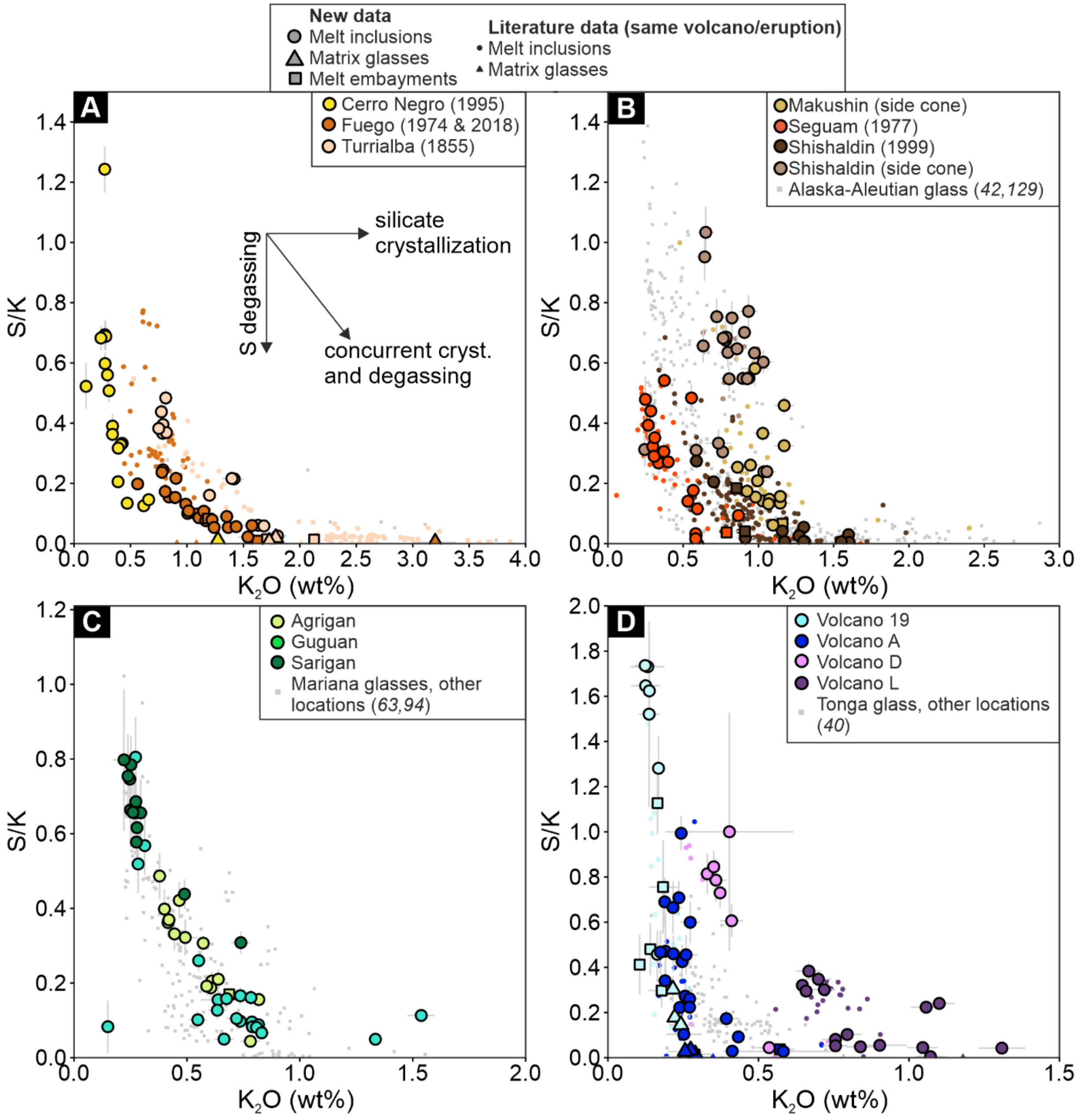

Figure S4: The composition of analyzed glasses on S/K vs. K_2O (wt%) diagrams. Data from Central America (A), Aleutians (B), Marianas (C), and Tonga (D) are provided in separate plots. Arrows in (A) show approximate vectors of crystallization (increases K_2O at near-constant S/K), degassing (decreases S/K at constant K_2O), and concurrent crystallization and degassing (causes a negative correlation between S/K and K_2O). Symbols and literature references are the same as described in the caption of Figure S1.

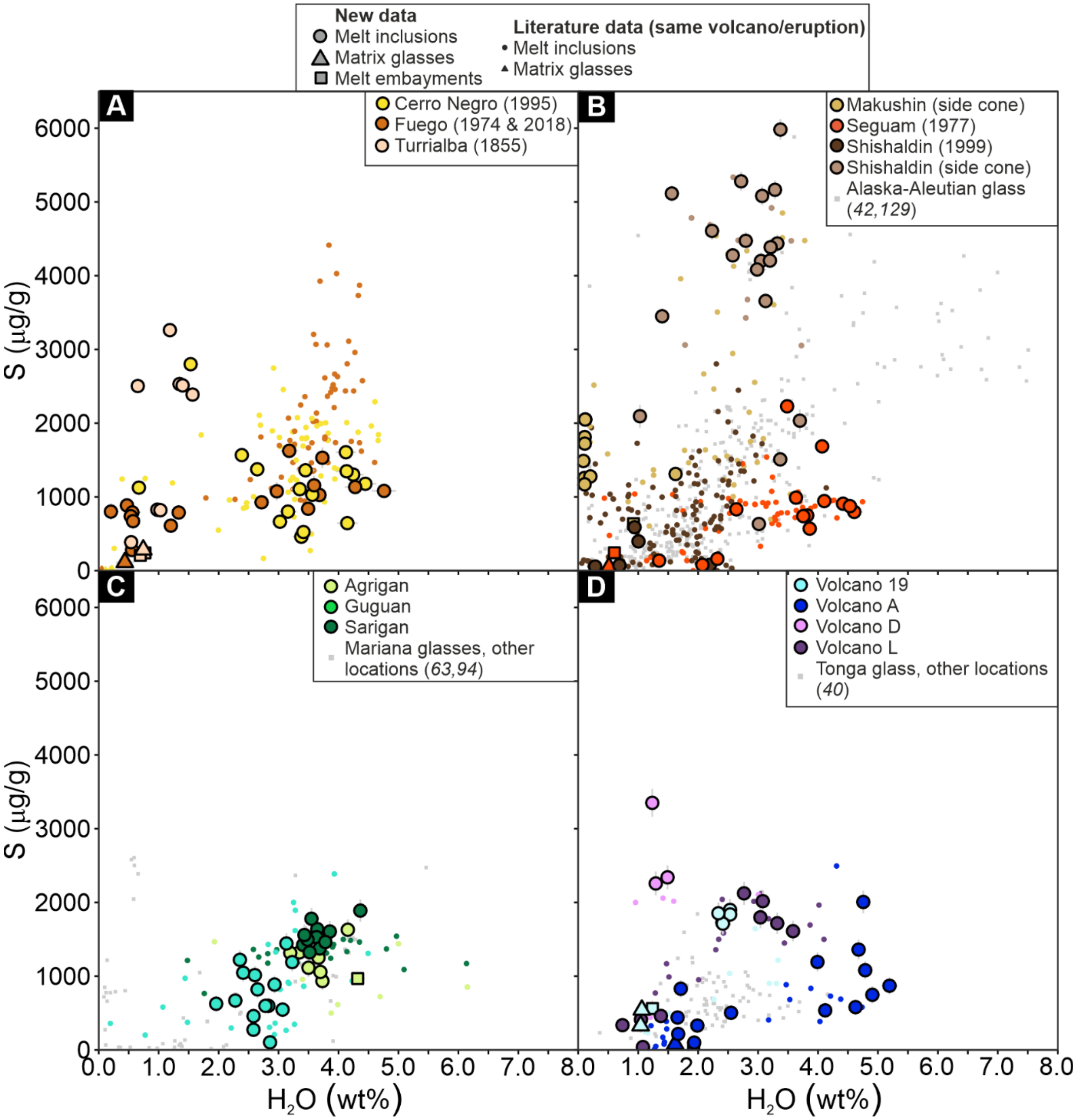

Figure S5: The composition of analyzed glasses on S (µg/g) vs. H_2O (wt%) diagrams. Data from Central America (A), Aleutians (B), Marianas (C), and Tonga (D) are provided in separate plots. Symbols and literature references are the same as described in the caption of Figure S1.

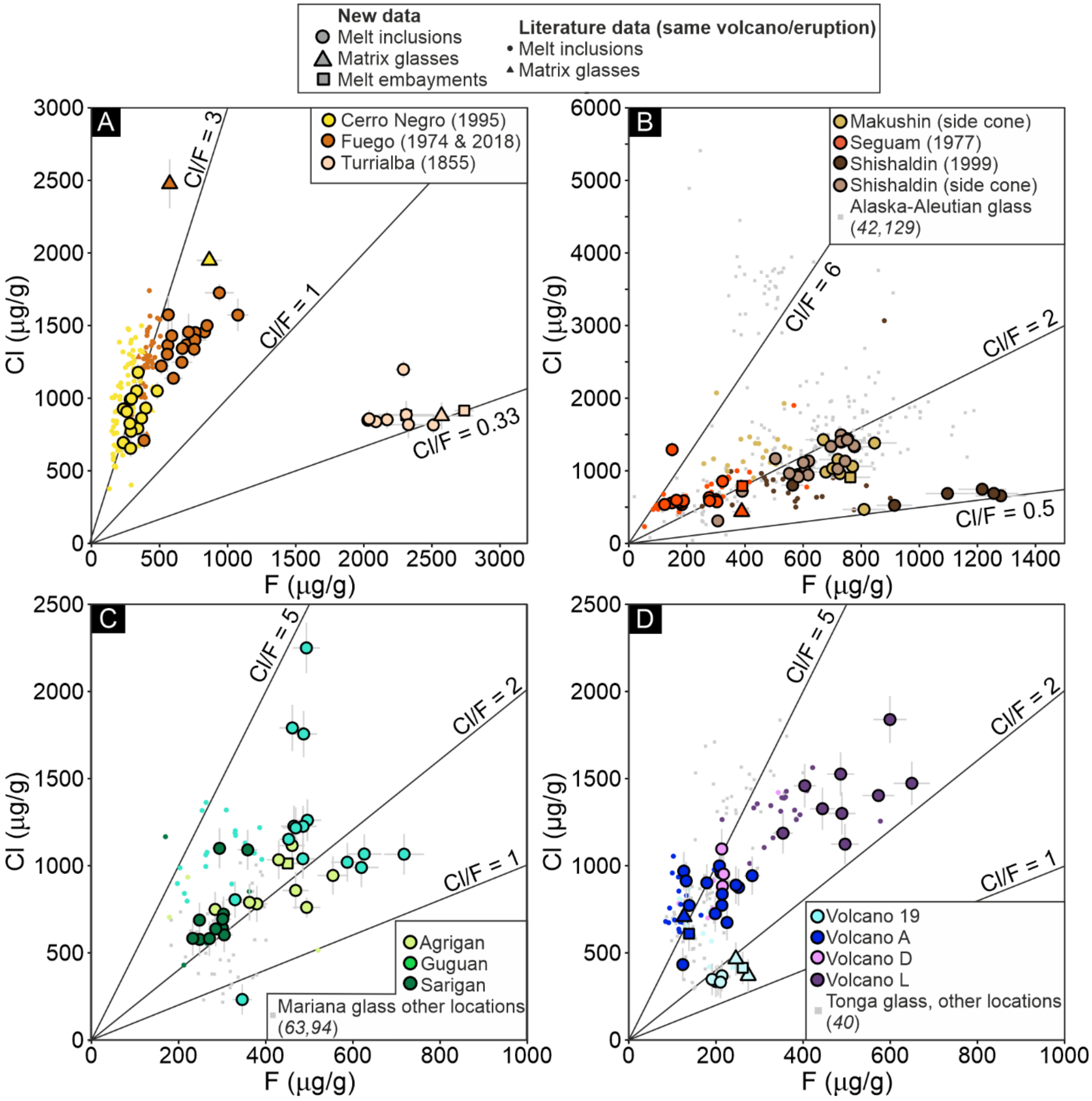

Figure S6: Chlorine and Fluorine contents of MIs from the four studied arcs. Data from Central America (A), Aleutians (B), Marianas (C), and Tonga (D) are provided in separate plots. Symbols are the same as in Figure S2. Literature data are taken from Barth et al. (*132*) and Rasmussen et al. (*128*) for Central America and Rasmussen and Plank (*42*) for the Aleutians. In (C), literature data from the Marians is presented (*63,94*). In (D) literature data for Tonga is from Cooper et al. (*40*). Grey squares are data from other volcanoes from the same arcs. Error bars are 1σ.

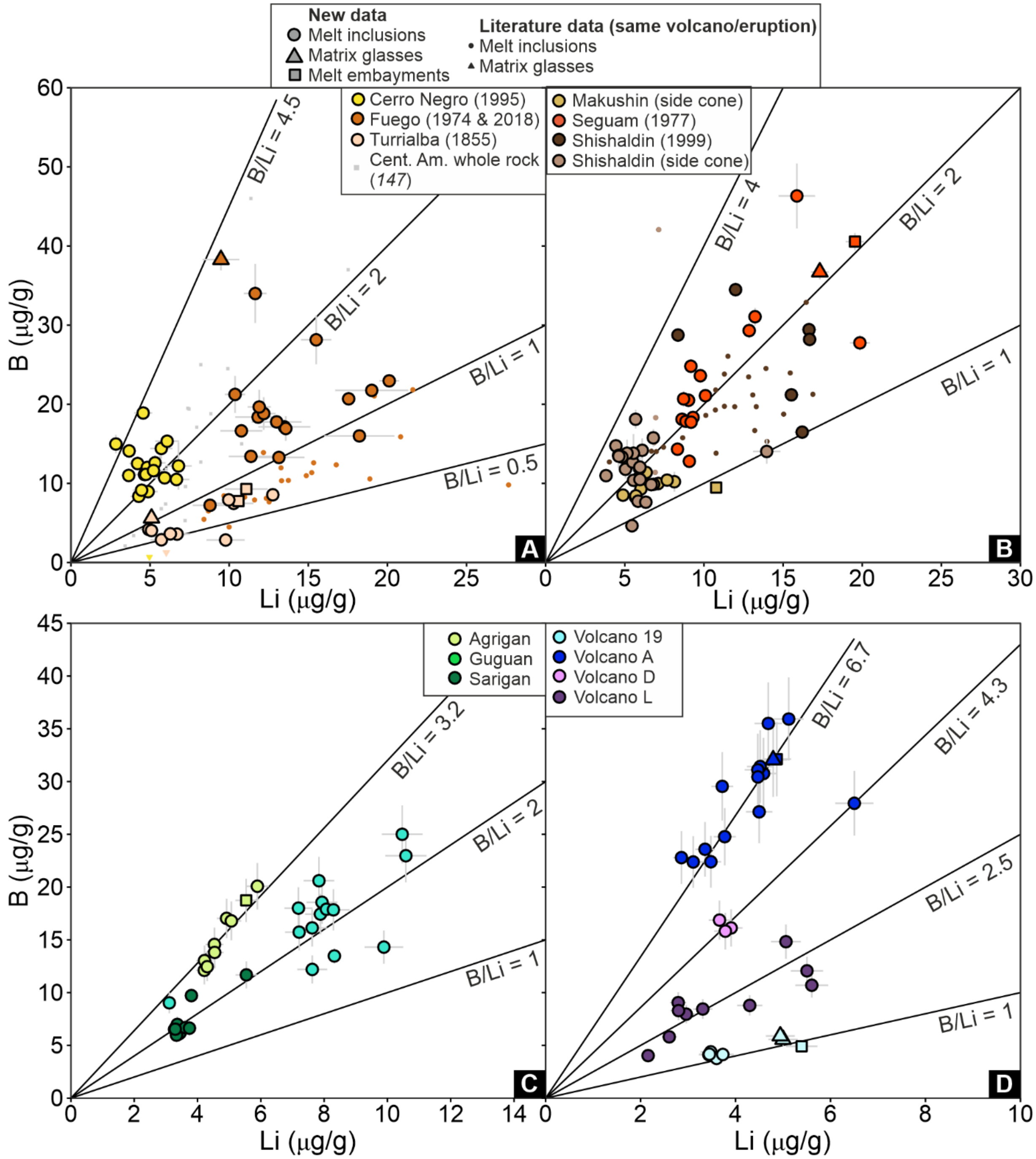

Figure S7: Boron versus lithium contents in the studied glasses. Data from Central America (A), Aleutians (B), Marianas (C), and Tonga (D) are provided in separate plots. Literature data for Central America whole-rock samples are from Carr et al. (*147*); Aleutian data is taken from Rasmussen and Plank (*42*). Error bars are 1σ.

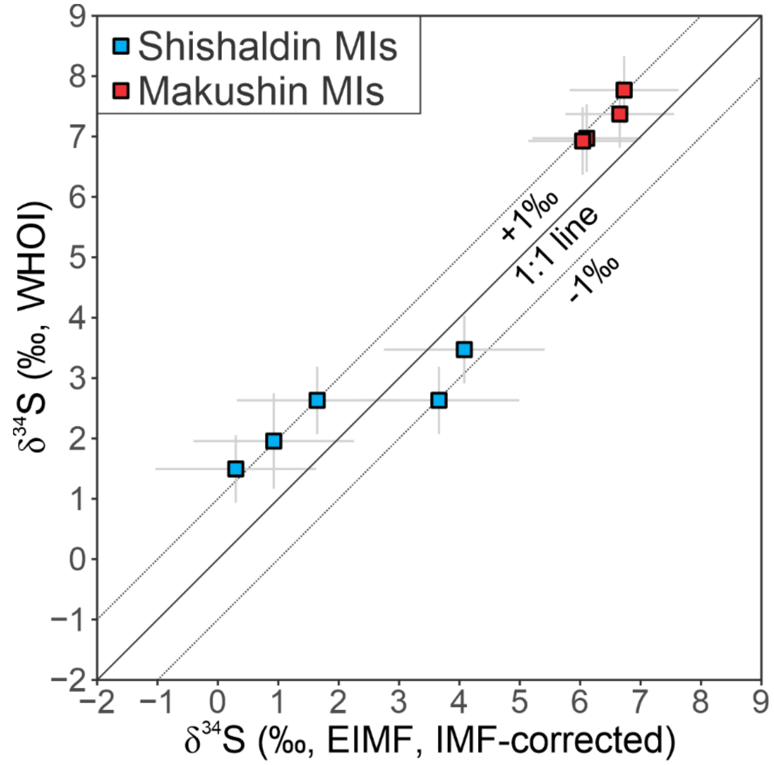

Figure S8: Interlaboratory comparison of sulfur isotope analyses. Sulfur isotope ratios measured from selected Shishaldin (blue) and Makushin (red) at WHOI and Edinburgh. Data collected in Edinburgh was corrected using a ln(S) regression to account for the sulfur content-dependent IMF observed between different standards. Error bars are 1σ.

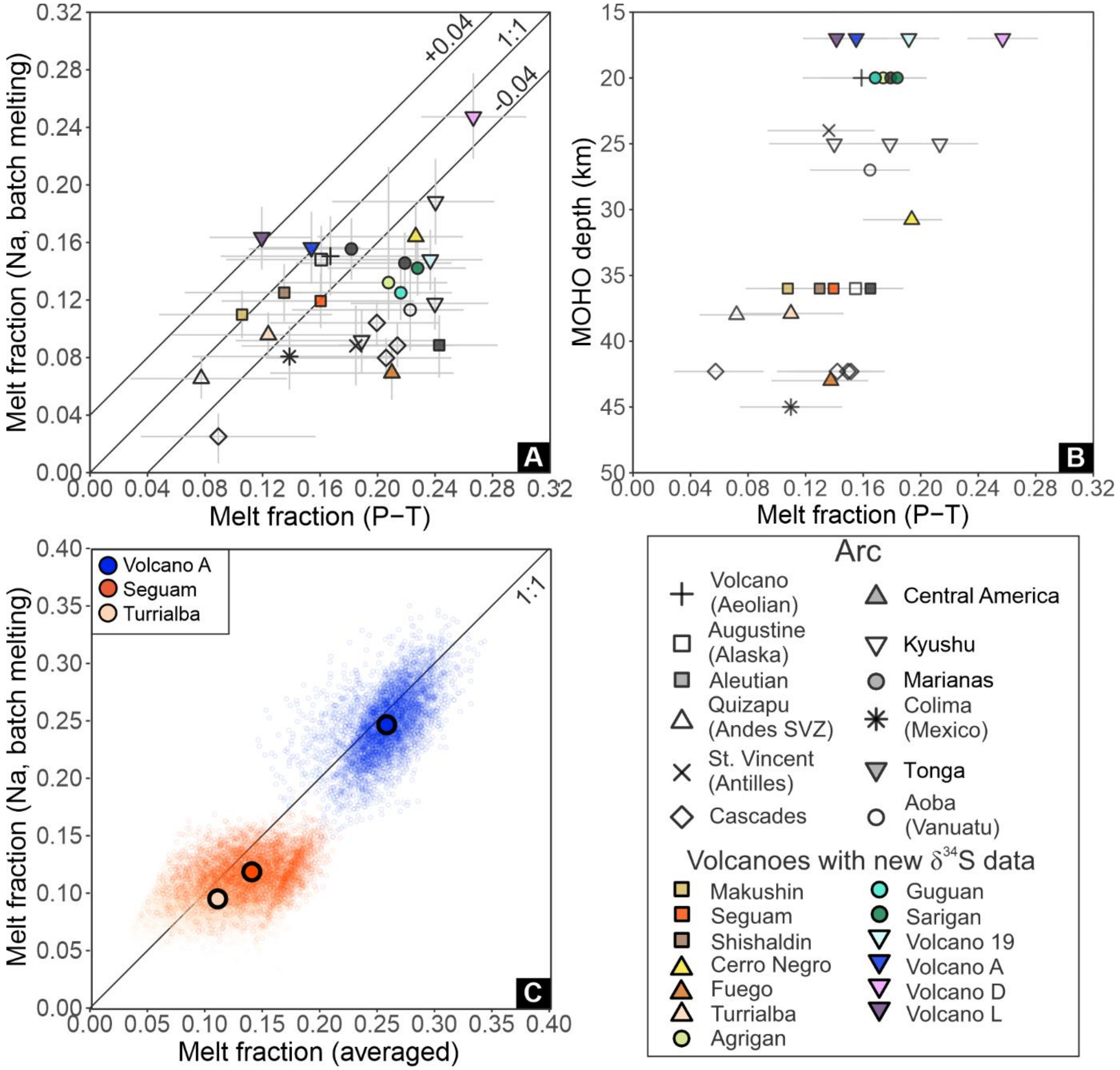

Figure S9: Melting degree estimates for our global arc magma dataset. In (A) two independent melting estimates (Na content and thermobarometry-based) are plotted against each other. In (B) crustal thickness is plotted against melt fraction, calculated using our P-T approach. Similar to fractionation-corrected global arc segment Na contents (*95*), there is a broad correlation between our melt fraction estimates for each volcanic system and crustal thickness. (C) Results of our Monte Carlo simulations for Seguam, Turrialba and volcano A from Tonga showing Na-content based melting degree against averaged melting degree. Larger circles show the median melt fraction values for the three different volcanic systems. Unfilled circles are individual outcomes and represent uncertainty. Error bars in (A) and (B) are 70% intervals taken from the Monte Carlo simulation.

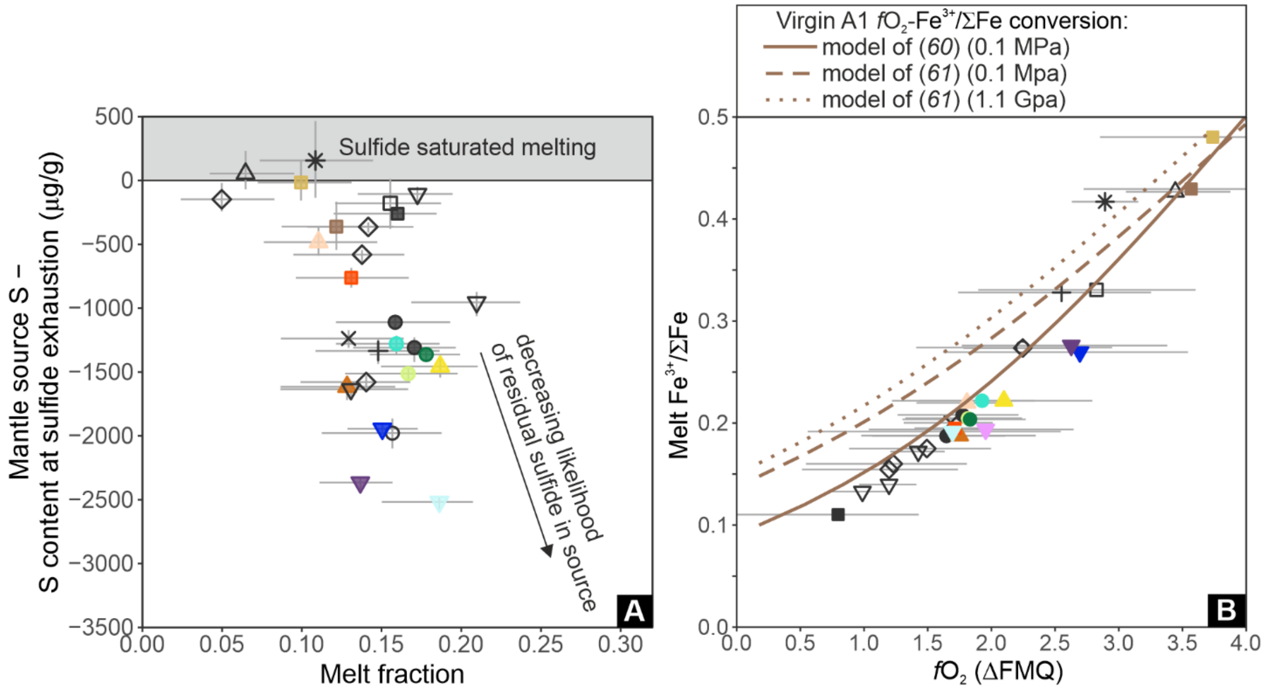

Figure S10: Further details on sulfide exhaustion and fO_2 models used in the main text. (A) Calculated difference between mantle wedge S content estimates presented in Figure 6 in the main text, and expected mantle sulfur contents at sulfide exhaustion, which is calculated by multiplying primary melt total S solubility with F. Grey area indicates the field in which residual sulfide in the mantle is expected. (B) Iron speciation of volcanic systems included in this study plotted against fO_2 relative to the FMQ buffer. To compare conversion models, iron speciation as a function of fO_2 was calculated for sample Virgin A1 (Virgin Cone, Shishaldin) at 1184 °C. The solid line shows our preferred model (*60*), while the dotted line shows the commonly used model of Kress and Carmichael (*61*) at 0.1 MPa, while the dashed line shows the result of the same model at 1.1 GPa. Error bars are 70% confidence intervals.

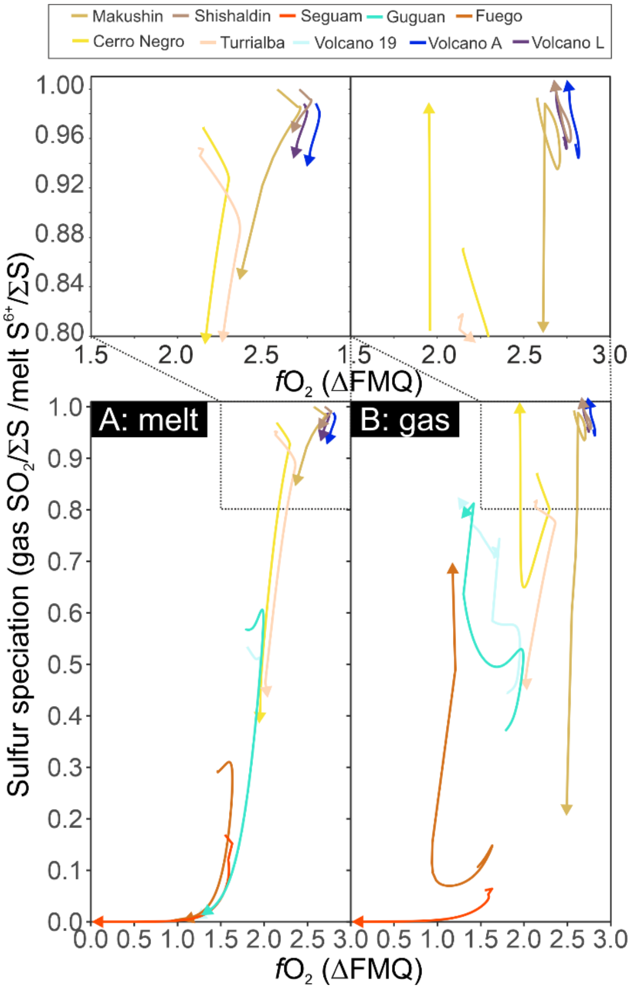

Figure S11: Modelled sulfur speciation melts and gases. We calculate sulfur speciation ($SO_2/\Sigma S$ and $S^{6+}/\Sigma S$) for volcanic gases (A) and melts (B) during degassing modeled using Sulfur_X (*51*). Arrows pointing towards the direction of degassing. Top two figures highlight the area (high fO_2 degassing paths) indicated by the dotted lines in the two lower figures.

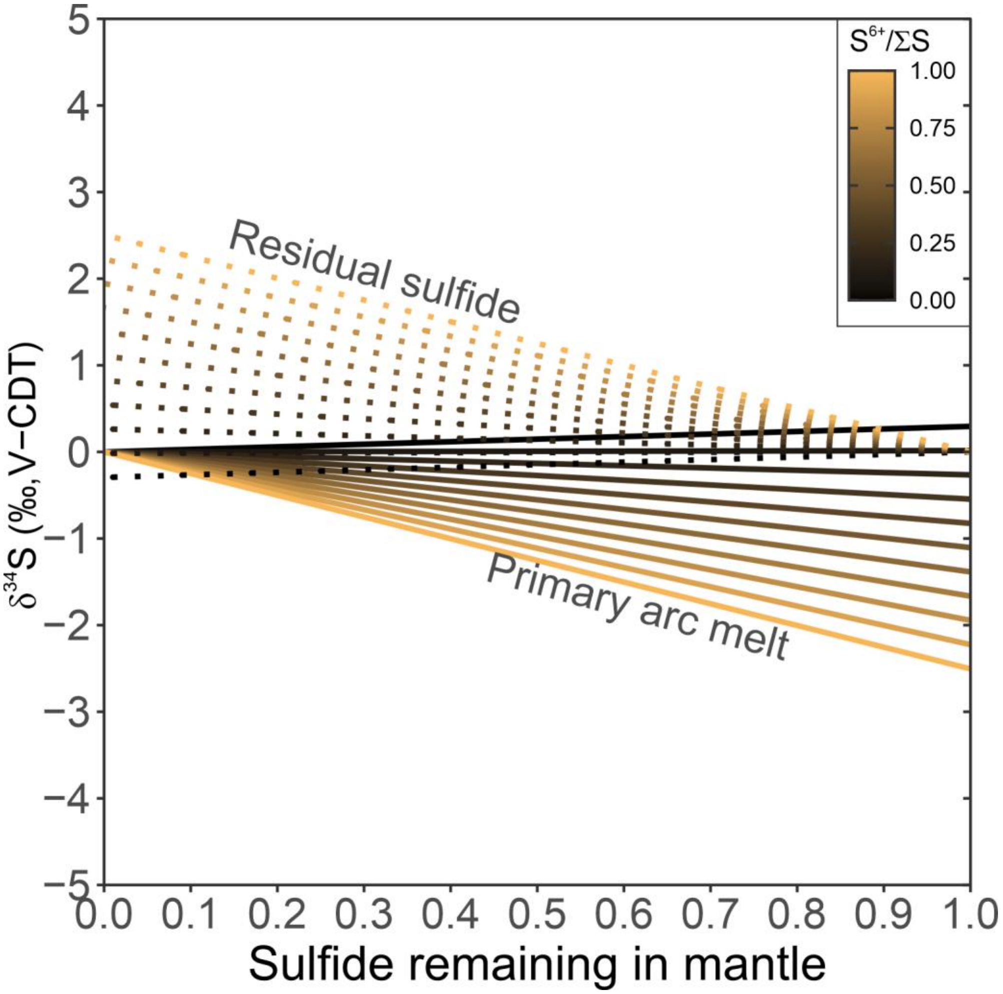

Figure S12: Sulfur isotope fractionation during sulfide fractionation. Equilibrium closed system melting models of sulfur isotope fractionation calculated at 1300°C, using equations presented in Marini et al. (*17*) and reference therein. Melt sulfur speciation varied from 0 to 1 in 0.1 increments, which is indicated by the color of each curve. Dashed lines show the modelled composition of residual sulfide as a fraction of remaining sulfur in the mantle, while solid lines show the sulfur isotope ratio of the melt.

Table S1: Important parameters taken from our Monte Carlo model. Median values of selected parameters taken from our Monte Carlo simulation for each locality in our dataset. Errors and more detailed results are provided in the supplementary spreadsheets.

Locality	Arc	Melting degree	melt H_2O (wt%)	mantle H_2O (wt%)	melt $Fe^{3+}/\Sigma Fe$	melt fO_2 (ΔFMQ)	mantle Fe_2O_3 (wt%)	melt S (µg/g)	mantle S (µg/g)	Slab S fraction in mantle	mantle $\delta^{34}S$ (‰)	slab component $\delta^{34}S$ (‰)
**Agrigan**	**Marianas**	**17.4%**	**3.79**	**0.686**	**0.204**	**1.8**	**0.885**	**1551**	**267**	**44.9%**	**2.2**	**5.6**
Alamagan	Marianas	16.9%	3.77	0.657	0.208	1.7	0.812	1365	226	38.0%		
Aoba - Red Cliff	Vanuatu	16.5%	3.56	0.603	0.273	2.2	1.276	2615	420	62.5%		
Aso-Kishimadake	Kyushu	17.8%	2.40	0.438	0.133	1.0	0.634	2304	400	61.6%	7.1	11.5
Augustine	Alaska	15.4%	6.59	1.069	0.330	2.7	1.350	5664	866	83.3%		
**Cerro Negro (1995)**	**Central America**	**19.4%**	**3.83**	**0.758**	**0.222**	**2.0**	**1.004**	**2350**	**444**	**65.5%**	**1.6**	**2.9**
Colima	Mexico	11.0%	6.30	0.756	0.416	2.9	1.479	8359	920	83.2%		
**Fuego**	**Central America**	**13.8%**	**3.60**	**0.510**	**0.188**	**1.7**	**0.861**	**2489**	**329**	**53.6%**	**3.3**	**6.7**
Fukue	Kyushu	13.9%	1.64	0.236	0.172	1.4	0.795	1166	157	27.6%	1.7	8.7
**Guguan**	**Marianas**	**16.8%**	**3.57**	**0.616**	**0.223**	**1.9**	**0.956**	**2081**	**340**	**54.4%**	**3.6**	**7.0**
Lassen-BBL	Cascades	14.3%	1.03	0.151	0.154	1.1	0.660	1308	178	31.1%	1.7	7.4
Lassen-BPPC	Cascades	15.2%	2.50	0.391	0.160	1.2	0.581	1151	169	28.3%	3.5	15.3
Lassen-BRM	Cascades	5.7%	4.06	0.286	0.273	2.2	0.970	3768	224	43.5%	5.2	12.3
Lassen-BRVB	Cascades	15.0%	1.74	0.265	0.174	1.4	0.754	1748	250	42.3%	4.1	10.5
**Makushin (Pakushin cone)**	**Aleutian**	**10.8%**	**3.53**	**0.421**	**0.480**	**3.7**	**1.967**	**5202**	**567**	**72.1%**	**4.6**	**6.6**
Pagan	Marianas	17.9%	4.63	0.847	0.188	1.6	0.788	2863	498	69.2%		
Quizapu - Los Hornitos	Andes SVZ	7.2%	3.28	0.282	0.426	3.5	1.590	5311	399	59.0%		
**Sarigan**	**Marianas**	**18.4%**	**4.04**	**0.761**	**0.203**	**1.8**	**0.868**	**1662**	**298**	**49.1%**	**3.6**	**7.9**
**Seguam (1977)**	**Aleutian**	**14.0%**	**4.11**	**0.611**	**0.195**	**1.7**	**0.726**	**2179**	**303**	**50.0%**	**4.2**	**8.9**
**Shishaldin (1999)**	**Aleutian**	**16.5%**	**1.68**	**0.285**	**0.109**	**0.6**	**0.577**	**1234**	**198**	**33.3%**		
**Shishaldin (Virigin cone)**	**Aleutian**	**13.0%**	**3.59**	**0.502**	**0.426**	**3.5**	**2.025**	**5310**	**692**	**77.7%**	**2.4**	**3.2**
St Vincent	Antilles	13.5%	5.00	0.708	0.200	1.7	0.790	1704	225	39.2%		
Sumiyoshi-ike	Kyushu	21.3%	0.97	0.211	0.140	1.2	0.736	2644	552	72.6%	3.7	5.4
**Turrialba (1865)**	**Central America**	**11.0%**	**1.53**	**0.185**	**0.220**	**1.8**	**0.831**	**3102**	**343**	**54.4%**	**1.2**	**2.7**
**Volcano 19**	**Tonga**	**19.2%**	**2.75**	**0.542**	**0.193**	**1.5**	**0.885**	**1604**	**302**	**49.9%**	**3.3**	**7.2**
**Volcano A**	**Tonga**	**15.5%**	**4.21**	**0.692**	**0.267**	**2.5**	**1.121**	**1686**	**261**	**43.2%**	**0.9**	**3.2**
**Volcano D**	**Tonga**	**25.7%**	**2.29**	**0.603**	**0.196**	**1.9**	**1.054**	**2154**	**552**	**73.2%**	**1.7**	**2.5**
**Volcano L**	**Tonga**	**14.2%**	**3.38**	**0.505**	**0.275**	**2.5**	**1.187**	**1915**	**268**	**44.5%**	**3.4**	**8.5**
Vulcano	Aeolian	15.9%	3.71	0.615	0.328	2.5	1.597	2563	401	60.7%		

Supplementary file caption: Excel spreadsheets containing data and model results. Spreadsheet S1 contains glass data presented in the paper. Explanation for each column is provided at top of the spreadsheet. Spreadsheet S2 contains the summary of important parameters used in the model calculations for the localities in our global melt composition data compilation. Spreadsheet S3 contains melt compositions used for primary melt and melting degree estimation. Spreadsheet S4 shows the summarized results of the Monte Carlo simulations. Spreadsheet S5 and S6 contains unprocessed and processed sulfur isotope data collected in Edinburgh (EIMF) and Woods Hole (WHOI) SIMS laboratories. Spreadsheets S7 and S8 present unprocessed trace element and CO_2 SIMS data collected at EIMF in 2024, respectively. Spreadsheet S9 includes our binary DMM-slab component mixing model results presented in the main text (Figure 6). Spreadsheet S10 presents the results of the Sulfur_X degassing models shown in Figures 1 and 2.

REFERENCES

1. E. Médard, T. L. Grove, The effect of H_2O on the olivine liquidus of basaltic melts: experiments and thermodynamic models. *Contrib. Mineral. Petrol.* **155**, 417–432 (2008).

2. T. J. Tenner, M. M. Hirschmann, M. Humayun, The effect of H_2O on partial melting of garnet peridotite at 3.5 GPa. *Geochem. Geophys. Geosyst.* **13**, Q03016 (2012).

3. T. L. Grove, C. B. Till, M. J. Krawczynski, The role of H_2O in subduction zone magmatism. *Annu. Rev. Earth Planet. Sci.* **40**, 413–439 (2012).

4. M. M. Zimmer, T. Plank, E. H. Hauri, G. M. Yogodzinski, P. Stelling, J. Larsen, B. Singer, B. Jicha, C. Mandeville, C. J. Nye, The role of water in generating the calc-alkaline trend: New volatile data for Aleutian magmas and a new tholeiitic index. *J. Petrol.* **51**, 2411–2444 (2010).

5. C. Huber, M. Townsend, W. Degruyter, O. Bachmann, Optimal depth of subvolcanic magma chamber growth controlled by volatiles and crust rheology. *Nat. Geosci.* **12**, 762–768 (2019).

6. D. J. Rasmussen, T. A. Plank, D. C. Roman, M. M. Zimmer, Magmatic water content controls the pre-eruptive depth of arc magmas. *Science* **375**, 1169–1172 (2022).

7. C. Grondahl, Z. Zajacz, Sulfur and chlorine budgets control the ore fertility of arc magmas. *Nat. Commun.* **13**, 4218 (2022).

8. J. D. Devine, H. Sigurdsson, A. N. Davis, S. Self, Estimates of sulfur and chlorine yield to the atmosphere from volcanic eruptions and potential climatic effects. *J. Geophys. Res. Solid Earth* **89**, 6309–6325 (1984).

9. R. Dasgupta, M. M. Hirschmann, The deep carbon cycle and melting in Earth's interior. *Earth Planet. Sci. Lett.* **298**, 1–13 (2010).

10. N. Métrich, V. Zanon, L. Créon, A. Hildenbrand, M. Moreira, F. O. Marques, Is the Azores hotspot a wetspot? Insights from the geochemistry of fluid and melt inclusions in olivine of Pico basalts. *J. Petrol.* **55**, 377–393 (2014).

11. Z. Taracsák, M. E. Hartley, R. Burgess, M. Edmonds, M.-A. Longpré, B. D. Monteleone, R. Tartèse, A. V. Turchyn, The origin of sulfur in Canary Island magmas and its implications for Earths deep sulfur cycle. *Proc. Natl. Acad. Sci U.S.A.* **122**, e2416070122 (2025).

12. D. E. Canfield, The evolution of the Earth surface sulfur reservoir. *Am. J. Sci.* **304**, 839–861 (2004).

13. P. J. Jugo, M. Wilke, R. E. Botcharnikov, Sulfur K-edge XANES analysis of natural and synthetic basaltic glasses: Implications for S speciation and S content as function of oxygen fugacity. *Geochim. Cosmochim. Acta* **74**, 5926–5938 (2010).

14. H. S. O'Neill, J. A. Mavrogenes, The sulfate capacities of silicate melts. *Geochim. Cosmochim. Acta* **334**, 368–382 (2022).

15. M. J. Muth, E. Cottrell, The effect of pressure on sulfur valence state in mafic silicate melts. *Earth Planet. Sci. Lett.* **668**, 119562 (2025).

16. J. E. Mungall, Roasting the mantle: Slab melting and the genesis of major Au and Au-rich Cu deposits. *Geology* **30**, 915–918 (2002).

17. L. Marini, R. Moretti, M. Accornero, Sulfur isotopes in magmatic hydrothermal systems, melts, and magmas. *Rev. Mineral. Geochem.* **73**, 423–492 (2011).

18. M. Wadhwa, Redox conditions on small bodies, the Moon and Mars. *Rev. Mineral. Geochem.* **68**, 493–510 (2008).

19. R. W. Thomas, B. J. Wood, Sulfur speciation in silicate melts at high pressure. *Geochim. Cosmochim. Acta* **417**, 37–51 (2026).

20. M. J. Muth, P. J. Wallace, Sulfur recycling in subduction zones and the oxygen fugacity of mafic arc magmas. *Earth Planet. Sci. Lett.* **599**, 117836 (2022).

21. A. G. Tomkins, K. A. Evans, Separate zones of sulfate and sulfide release from subducted mafic oceanic crust. *Earth Planet. Sci. Lett.* **428**, 73–83 (2015).

22. A. Rielli, A. G. Tomkins, O. Nebel, M. Raveggi, H. Jeon, L. Martin, J. N. Ávila, Sulfur isotope and PGE systematics of metasomatised mantle wedge. *Earth Planet. Sci. Lett.* **497**, 181–192 (2018).

23. J. Walters, A. Cruz-Uribe, H. Marschall, Sulfur loss from subducted altered oceanic crust and implications for mantle oxidation. *Geochem. Perspect. Lett.* **13**, 36–41 (2020).

24. J. M. de Moor, T. P. Fischer, T. Plank, Constraints on the sulfur subduction cycle in Central America from sulfur isotope compositions of volcanic gases. *Chem. Geol.* **588**, 120627 (2022).

25. M. J. Muth, P. J. Wallace, Slab-derived sulfate generates oxidized basaltic magmas in the southern Cascade arc (California, USA). *Geology* **49**, 1177–1181 (2021).

26. M. Kawaguchi, K. T. Koga, E. Rose-Koga, K. Shimizu, T. Ushikubo, A. Yoshiasa, Sulfur isotope and trace element systematics in arc magmas: Seeing through the degassing via a melt inclusion study of Kyushu Island volcanoes, Japan. *J. Petrol.* **63**, egac061 (2022).

27. Z. Taracsák, T. Mather, S. Ding, T. Plank, M. Brounce, D. Pyle, A. Aiuppa, EIMF, Sulfur from the subducted slab dominates the sulfur budget of the mantle wedge under volcanic arcs. *Earth Planet. Sci. Lett.* **602**, 117948 (2023).

28. P. Beaudry, D. A. Sverjensky, Oxidized sulfur species in slab fluids as a source of enriched sulfur isotope signatures in arcs. *Geochem. Geophys. Geosyst.* **25**, e2024GC011542 (2024).

29. J.-L. Li, E. M. Schwarzenbach, T. John, J. J. Ague, F. Huang, J. Gao, R. Klemd, M. J. Whitehouse, X.-S. Wang, Uncovering and quantifying the subduction zone sulfur cycle from the slab perspective. *Nat. Commun.* **11**, 514 (2020).

30. A. Ren, Z. Wang, S. Aulbach, K. Zong, X. Wang, Z. Zou, Y. Shen, H. Cheng, Z. Hu, Z. Zhu, Subduction-related transfer of sulfur and chalcophile elements recorded in continental mantle wedge peridotites. *Geochim. Cosmochim. Acta* **398**, 11–28 (2025).

31. J. Alt, J. Burdett, Sulfur in Pacific deep-sea sediments (Leg 129) and implications for cycling of sediment in subduction zones. *Proc. O.D.P. Sci. Results* **129**, 283–294 (1992).

32. J. A. Padrón-Navarta, V. López Sánchez-Vizcaíno, M. D. Menzel, M. T. Gómez-Pugnaire, C. J. Garrido, Mantle wedge oxidation from deserpentinization modulated by sediment-derived fluids. *Nat. Geosci.* **16**, 268–275 (2023).

33. A. Maffeis, M. L. Frezzotti, J. A. D. Connolly, D. Castelli, S. Ferrando, Sulfur disproportionation in deep COHS slab fluids drives mantle wedge oxidation. *Sci. Adv.* **10**, 268–275 (2024).

34. A. Peccia, T. Plank, S. Ding, L. Bolge, A. Aiuppa, S. Vizzini, C. Tramati, Z. Taracsák, D. M. Pyle, T. A. Mather, Solving the sulfur isotope discrepancy in Central America. *Chem. Geol.* **691**, 122901 (2025).

35. J. C. Alt, W. C. Shanks, Microbial sulfate reduction and the sulfur budget for a complete section of altered oceanic basalts, IODP Hole 1256D (eastern Pacific). *Earth Planet. Sci. Lett.* **310**, 73–83 (2011).

36. J. C. Alt, E. M. Schwarzenbach, G. L. Früh-Green, W. C. Shanks III, S. M. Bernasconi, C. J. Garrido, L. Crispini, L. Gaggero, J. A. Padrón-Navarta, C. Marchesi, The role of serpentinites in cycling of carbon and sulfur: Seafloor serpentinization and subduction metamorphism. *Lithos* **178**, 40–54 (2013).

37. E. M. Syracuse, P. E. van Keken, G. A. Abers, The global range of subduction zone thermal models. *Phys. Earth Planet. In.* **183**, 73–90 (2010).

38. R. D. Müller, M. Sdrolias, C. Gaina, W. R. Roest, Age, spreading rates, and spreading asymmetry of the world's ocean crust. *Geochem. Geophys. Geosyst.* **9**, 2007GC001743 (2008).

39. M. J. Le Bas, R. W. Le Maitre, A. Streckeisen, B. Zanettin, IUGS Subcommission on the Systematics of Igneous Rocks, A chemical classification of volcanic rocks based on the total alkali-silica diagram. *J. Petrol.* **27**, 745–750 (1986).

40. L. Cooper, T. Plank, R. Arculus, E. Hauri, K. A. Kelley, Arc backarc exchange along the Tonga-Lau system: Constraints from volatile elements. *J. Petrol.* **63**, egac072 (2022).

41. T. Plank, K. A. Kelley, M. M. Zimmer, E. H. Hauri, P. J. Wallace, Why do mafic arc magmas contain 4wt% water on average? *Earth Planet. Sci. Lett.* **364**, 168–179 (2013).

42. D. J. Rasmussen, T. A. Plank, Melt inclusion data for the central-eastern Aleutian volcanoes, Interdisciplinary Earth Data Alliance, Version 1.0. [accessed 30 October 2024] (2021).

43. G. A. Gaetani, J. A. OLeary, N. Shimizu, C. E. Bucholz, M. Newville, Rapid re-equilibration of H_2O and oxygen fugacity in olivine-hosted melt inclusions. *Geology* **40**, 915–918 (2012).

44. E. Ranta, J. Gunnarsson-Robin, S. A. Halldórsson, S. Ono, G. Izon, M. G. Jackson, C. D. Reekie, F. E. Jenner, G. H. Guðfinnsson, Ó. P. Jónsson, A. Stefánsson, Ancient and recycled sulfur sampled by the iceland mantle plume. *Earth Planet. Sci. Lett.* **584**, 117452 (2022).

45. J. Labidi, P. Cartigny, J. Birck, N. Assayag, J. Bourrand, Determination of multiple sulfur isotopes in glasses: A reappraisal of the MORB $\delta^{34}S$. *Chem. Geol.* **334**, 189–198 (2012).

46. J. Labidi, P. Cartigny, C. Hamelin, M. Moreira, L. Dosso, Sulfur isotope budget (^{32}S, ^{33}S, ^{34}S and ^{36}S) in Pacific Antarctic ridge basalts: A record of mantle source heterogeneity and hydrothermal sulfide assimilation. *Geochim. Cosmochim. Acta* **133**, 47–67 (2014).

47. J. C. Alt, W. C. Shanks, M. C. Jackson, Cycling of sulfur in subduction zones: The geochemistry of sulfur in the Mariana Island Arc and backarc trough. *Earth Planet. Sci. Lett.* **119**, 477–494 (1993).

48. E. C. Hughes, P. Liggins, L. Saper, E. M. Stolper, The effects of oxygen fugacity and sulfur on the pressure of vapor-saturation of magma. *Am. Mineral.* **109**, 422–438 (2024).

49. J. Boulliung, B. J. Wood, Sulfur oxidation state and solubility in silicate melts. *Contrib. Mineral. Petrol.* **178**, 56 (2023).

50. L. Marini, A. Paiotti, C. Principe, G. Ferrara, R. Cioni, Isotopic ratio and concentration of sulfur in the undersaturated alkaline magmas of Vulture Volcano (Italy). *Bull. Volcanol.* **56**, 487–492 (1994).

51. S. Ding, T. Plank, P. J. Wallace, D. J. Rasmussen, Sulfur_X: A model of sulfur degassing during magma ascent. *Geochem. Geophys. Geosyst.* **24**, e2022GC010552 (2023).

52. H. Sakai, T. J. Casadevall, J. G. Moore, Chemistry and isotope ratios of sulfur in basalts and volcanic gases at Kilauea Volcano, Hawaii. *Geochim. Cosmochim. Acta* **46**, 729–738 (1982).

53. L. Marini, V. Chiappini, R. Cioni, G. Cortecci, E. Dinelli, C. Principe, G. Ferrara, Effect of degassing on sulfur contents and $\delta^{34}S$ values in Somma-Vesuvius magmas. *Bull. Volcanol.* **60**, 187–194 (1998).

54. A. Fiege, F. Holtz, N. Shimizu, C. W. Mandeville, H. Behrens, J. L. Knipping, Sulfur isotope fractionation between fluid and andesitic melt: An experimental study. *Geochim. Cosmochim. Acta* **142**, 501–521 (2014).

55. S. Caliro, G. Chiodini, R. Avino, A. Carandente, E. Cuoco, M. Di Vito, C. Minopoli, F. Rufino, A. Santi, J. Lages, A. Mangiacapra, B. Monteleone, L. Pappalardo, Z. Taracsák, C. Tramati, S. Vizzini, A. Aiuppa, Escalation of caldera unrest indicated by increasing emission of isotopically light sulfur. *Nat. Geosci.* **18**, 167–174 (2025).

56. D. Milidragovic, J. A. Nott, D. W. Spence, D. Schumann, J. S. Scoates, G. T. Nixon, R. A. Stern, Sulfate recycling at subduction zones indicated by sulfur isotope systematics of Mesozoic ultramafic island arc cumulates in the North American Cordillera. *Earth Planet. Sci. Lett.* **620**, 118337 (2023).

57. S. Prabha-Mohan, K. T. Koga, D. F. Narváez, E. F. Rose-Koga, J. Labidi, Experimental determination of equilibrium sulfur isotope fractionation factors in the gas-silicate melt-sulfide liquid system. *Geochim. Cosmochim. Acta* **413**, 187–203 (2025).

58. E. J. Nicholson, P. E. Wieser, M. E. Hartley, F. E. Jenner, B. E. Kunz, E. Ilyinskaya, T. Thordarson, M. Edmonds, Sulfide saturation and resorption modulates sulfur and metal availability during the 2014–15 Holuhraun eruption, Iceland. *Commun. Earth Environ.* **5**, 164 (2024).

59. L. V. Danyushevsky, A. W. McNeill, A. V. Sobolev, Experimental and petrological studies of melt inclusions in phenocrysts from mantle-derived magmas: An overview of techniques, advantages and complications. *Chem. Geol.* **183**, 5–24 (2002).

60. H. S. C. O'Neill, A. J. Berry, G. Mallmann, The oxidation state of iron in Mid-Ocean Ridge Basaltic (MORB) glasses: Implications for their petrogenesis and oxygen fugacities. *Earth Planet. Sci. Lett.* **504**, 152–162 (2018).

61. V. C. Kress, I. S. E. Carmichael, The compressibility of silicate liquids containing Fe_2O_3 and the effect of composition, temperature, oxygen fugacity and pressure on their redox states. *Contrib. Mineral. Petrol.* **108**, 82–92 (1991).

62. A. K. Matzen, A. Woodland, J. R. Beckett, B. J. Wood, Oxidation state of iron and Fe-Mg partitioning between olivine and basaltic martian melts. *Am. Mineral.* **107**, 1442–1452 (2022).

63. M. N. Brounce, K. A. Kelley, E. Cottrell, Variations in $Fe^{3+}/\Sigma Fe$ of Mariana arc basalts and mantle wedge fO_2. *J. Petrol.* **55**, 2513–2536 (2014).

64. T. A. Shishkina, M. V. Portnyagin, R. E. Botcharnikov, R. R. Almeev, A. V. Simonyan, D. Garbe-Schönberg, S. Schuth, M. Oeser, F. Holtz, Experimental calibration and implications of olivine-melt vanadium oxybarometry for hydrous basaltic arc magmas. *Am. Mineral.* **103**, 369–383 (2018).

65. S. Erdmann, M. Pichavant, F. Gaillard, Mineral-melt vanadium oxybarometry for primitive arc magmas: Effect of hydrous melt composition on fO_2 estimates. *Contrib. Mineral. Petrol.* **179**, 39 (2024).

66. Y. Moussallam, G. Georgeais, S. Ding, J.-L. Devidal, B. Scaillet, C. Oppenheimer, A. Burgisser, E. Rose-Koga, K. Koga, N. Peters, A. Peccia, P. Samaniego, N. Métrich, P. Robidoux, M. Kawaguchi, On the oxidation state of arc magmas. *Geochem. Perspect. Lett.* **39**, 42–47 (2026).

67. M. E. Hartley, O. Shorttle, J. Maclennan, Y. Moussallam, M. Edmonds, Olivine-hosted melt inclusions as an archive of redox heterogeneity in magmatic systems. *Earth Planet. Sci. Lett.* **479**, 192–205 (2017).

68. Y. Moussallam, C. Oppenheimer, B. Scaillet, F. Gaillard, P. Kyle, N. Peters, M. Hartley, K. Berlo, A. Donovan, Tracking the changing oxidation state of Erebus magmas, from mantle to surface, driven by magma ascent and degassing. *Earth Planet. Sci. Lett.* **393**, 200–209 (2014).

69. M. Brounce, E. Stolper, J. Eiler, Redox variations in Mauna Kea lavas, the oxygen fugacity of the Hawaiian plume, and the role of volcanic gases in Earths oxygenation. *Proc. Natl. Acad. Sci. U.S.A.* **114**, 8997–9002 (2017).

70. Y. Moussallam, M. Edmonds, B. Scaillet, N. Peters, E. Gennaro, I. Sides, C. Oppenheimer, The impact of degassing on the oxidation state of basaltic magmas: A case study of Kīlauea volcano. *Earth Planet. Sci. Lett.* **450**, 317–325 (2016).

71. M.-A. Longpré, J. Stix, A. Klügel, N. Shimizu, Mantle to surface degassing of carbon- and sulphur-rich alkaline magma at El Hierro, Canary Islands. *Earth Planet. Sci. Lett.* **460**, 268–280 (2017).

72. K. A. Kelley, E. Cottrell, Water and the oxidation state of subduction zone magmas. *Science* **325**, 605–607 (2009).

73. M. Gaborieau, M. Laubier, M. Pompilio, N. Bolfan-Casanova, Determination of the oxidation state of primary melts using two proxies. *Chem. Geol.* **638**, 121701 (2023).

74. E. C. Hughes, L. M. Saper, P. Liggins, H. S. C. O'Neill, E. M. Stolper, The sulfur solubility minimum and maximum in silicate melt. *J. Geol. Soc. London* **180**, jgs2021-125 (2023).

75. E. Hughes, P. Liggins, P. Wieser, E. Stolper, VolFe: An open-source Python package for calculating melt-vapour equilibria including silicate melt, carbon, hydrogen, sulfur, and noble gases. *Volcanica* **8**, 457481 (2025).

76. K. A. Kelley, E. Cottrell, The influence of magmatic differentiation on the oxidation state of Fe in a basaltic arc magma. *Earth Planet. Sci. Lett.* **329–330**, 109–121 (2012).

77. S. Tassara, M. Reich, C. Cannatelli, B. Konecke, D. Kausel, D. Morata, F. Barra, A. Simon, A. Fiege, E. Morgado, M. Leisen, Post-melting oxidation of highly primitive basalts from the southern Andes. *Geochim. Cosmochim. Acta* **273**, 291–312 (2020).

78. C.-T. A. Lee, P. Luffi, T. Plank, H. Dalton, W. P. Leeman, Constraints on the depths and temperatures of basaltic magma generation on Earth and other terrestrial planets using new thermobarometers for mafic magmas. *Earth Planet. Sci. Lett.* **279**, 20–33 (2009).

79. C. Herzberg, P. D. Asimow, PRIMELT3 MEGA.XLSM software for primary magma calculation: Peridotite primary magma MgO contents from the liquidus to the solidus. *Geochem. Geophys. Geosyst.* **16**, 563–578 (2015).

80. M. Carr, M. Feigenson, L. Patino, J. Walker, Volcanism and geochemistry in Central America: Progress and problems. *Geophys. Monogr. Ser.* **138**, 153–174 (2003).

81. V. J. M. Salters, A. Stracke, Composition of the depleted mantle. *Geochem. Geophys. Geosyst.* **5**, 2003GC000597 (2004).

82. S. J. Turner, C. H. Langmuir, Sediment and ocean crust both melt at subduction zones. *Earth Planet. Sci. Lett.* **584**, 117424 (2022).

83. C. Ballhaus, Redox states of lithospheric and asthenospheric upper mantle. *Contrib. Mineral. Petrol.* **114**, 331–348 (1993).

84. K. D. Putirka, Thermometers and barometers for volcanic systems. *Rev. Mineral. Geochem.* **69**, 61–120 (2008).

85. T. Plank, C. H. Langmuir, Tracing trace elements from sediment input to volcanic output at subduction zones. *Nature* **362**, 739–743 (1993).

86. K. A. Kelley, T. Plank, L. Farr, J. Ludden, H. Staudigel, Subduction cycling of U, Th, and Pb. *Earth Planet. Sci. Lett.* **234**, 369–383 (2005).

87. R. F. Katz, M. Spiegelman, C. H. Langmuir, A new parameterization of hydrous mantle melting. *Geochem. Geophys. Geosyst.* **4**, 1073 (2003).

88. D. H. Green, W. O. Hibberson, I. Kovacs, A. Rosenthal, Water and its influence on the lithosphere-asthenosphere boundary. *Nature* **467**, 448–451 (2010).

89. H. Keppler, Fluids and trace element transport in subduction zones. *Am. Mineral.* **102**, 5–20 (2017).

90. C. H. Langmuir, A. Bézos, S. Escrig, S. W. Parman, Chemical systematics and hydrous melting of the mantle in back-arc basins. *Geophys. Monogr. Ser.* **166**, 87–146 (2006).

91. E. R. Benjamin, T. Plank, J. A. Wade, K. A. Kelley, E. H. Hauri, G. E. Alvarado, High water contents in basaltic magmas from Irazú Volcano, Costa Rica. *J. Volcanol. Geotherm. Res.* **168**, 68–92 (2007).

92. K. Heydolph, K. Hoernle, F. Hauff, P. van den Bogaard, M. Portnyagin, I. Bindeman, D. Garbe-Schönberg, Along and across arc geochemical variations in NW Central America: Evidence for involvement of lithospheric pyroxenite. *Geochim. Cosmochim. Acta* **84**, 459–491 (2012).

93. M. M. Hirschmann, Mantle solidus: Experimental constraints and the effects of peridotite composition. *Geochem. Geophys. Geosyst.* **1**, 2000GC000070 (2000).

94. K. A. Kelley, T. Plank, S. Newman, E. M. Stolper, T. L. Grove, S. Parman, E. H. Hauri, Mantle melting as a function of water content beneath the Mariana arc. *J. Petrol.* **51**, 1711–1738 (2010).

95. S. J. Turner, C. H. Langmuir, The global chemical systematics of arc front stratovolcanoes: Evaluating the role of crustal processes. *Earth Planet. Sci. Lett.* **422**, 182–193 (2015).

96. T. Plank, C. H. Langmuir, The chemical composition of subducting sediment and its consequences for the crust and mantle. *Chem. Geol.* **145**, 325–394 (1998).

97. C. Sun, R. Dasgupta, Thermobarometry of CO_2-rich, silica- undersaturated melts constrains cratonic lithosphere thinning through time in areas of kimberlitic magmatism. *Earth Planet. Sci. Lett.* **550**, 116549 (2020).

98. J. Hermann, C. J. Spandler, Sediment melts at sub-arc depths: An experimental study. *J. Petrol.* **49**, 717–740 (2008).

99. K. Klimm, J. D. Blundy, T. H. Green, Trace element partitioning and accessory phase saturation during H_2O-saturated melting of basalt with implications for subduction zone chemical fluxes. *J. Petrol.* **49**, 523–553 (2008).

100. J. A. Pearce, D. W. Peate, Tectonic implications of the composition of volcanic arc magmas. *Annu. Rev. Earth Planet. Sci.* **23**, 251–285 (1995).

101. T. Sisson, S. Bronto, Evidence for pressure-release melting beneath magmatic arcs from basalt at Galunggung, India. *Nature* **391**, 883–886 (1998).

102. R. J. Stern, Subduction zones. *Rev. Geophys.* **40**, 3-1–3-38 (2002).

103. A. Rosenthal, E. H. Hauri, M. M. Hirschmann, Experimental determination of C, F, and H partitioning between mantle minerals and carbonated basalt, CO_2/Ba and CO_2/Nb systematics of partial melting, and the CO_2 contents of basaltic source regions. *Earth Planet. Sci. Lett.* **412**, 77–87 (2015).

104. F. A. Davis, E. Cottrell, Partitioning of Fe_2O_3 in peridotite partial melting experiments over a range of oxygen fugacities elucidates ferric iron systematics in mid-ocean ridge basalts and ferric iron content of the upper mantle. *Contrib. Mineral. Petrol.* **176**, 67 (2021).

105. C.-T. A. Lee, P. Luffi, E. J. Chin, R. Bouchet, R. Dasgupta, D. M. Morton, V. Le Roux, Q.-Z. Yin, D. Jin, Copper systematics in arc magmas and implications for crust-mantle differentiation. *Science* **336**, 64–68 (2012).

106. S.-Y. Zhao, A. Y. Yang, C. H. Langmuir, T.-P. Zhao, Oxidized primary arc magmas: Constraints from Cu/Zr systematics in global arc volcanics. *Sci. Adv.* **8**, eabk0718 (2022).

107. M.-A. Fortin, J. Riddle, Y. Desjardins-Langlais, D. R. Baker, The effect of water on the sulfur concentration at sulfide saturation (SCSS) in natural melts. *Geochim. Cosmochim. Acta* **160**, 100–116 (2015).

108. Z. Zajacz, A. Tsay, An accurate model to predict sulfur concentration at anhydrite saturation in silicate melts. *Geochim. Cosmochim. Acta* **261**, 288–304 (2019).

109. S. Ding, R. Dasgupta, The fate of sulfide during decompression melting of peridotite—Implications for sulfur inventory of the MORB-source depleted upper mantle. *Earth Planet. Sci. Lett.* **459**, 183–195 (2017).

110. R. Huang, H. Keppler, Anhydrite stability and the effect of Ca on the behavior of sulfur in felsic magmas. *Am. Mineral.* **100**, 257–266 (2015).

111. D. Rea, I. Basov, T. Janecek, A. Palmer-Julson, E. Arnold, J. A. Barron, J. F. Luc Beaufort, P. Bristow, Q. J. deMenocal, A. Y. Dubuisson, T. Qiadenokov, L. Hamilton, L. D. Ingram, R. A. Keigwin Jr, A. Keller L. A. Kotilainen, B. Krissek, J. J. McKelvey, M. Morley, Q. Okada, R. M. Olafsson, D. Owen, T. F. Pak, J. A. Pedersen, A. K. Roberts, V. V. Rutledge, H. Shilov, R. Snoeckx, R. Stax, R. Tiedemann, R. Weeks, *Proceedings of the Ocean Drilling Program Initial Reports* (Texas A&M University, Ocean Drilling Program, 1993), vol. 145.

112. J. Jaeger, S. Gulick, L. LeVay, H. Asahi, H. Bahlburg, C. Belanger, G. Berbel, L. Childress, E. Cowan, L. Drab, the Expedition 341 Scientists, "Expedition reports Southern Alaska margin," in *Proceedings of the Integrated Ocean Drilling Program* (Integrated Ocean Drilling Program, 2014), vol. 341.

113. S. D'Hondt, F. Inagaki, C. Alvarez Zarikian, the Expedition 329 Scientists, "Site U1365," in *Proceedings of the Integrated Ocean Drilling Program* (Integrated Ocean Drilling Program, 2014), vol. 329.

114. J. W. Dottin, J. Labidi, V. Lekic, M. G. Jackson, J. Farquhar, Sulfur isotope characterization of primordial and recycled sources feeding the Samoan mantle plume. *Earth Planet. Sci. Lett.* **534**, 116073 (2020).

115. R. A. Cabral, M. G. Jackson, E. F. Rose-Koga, K. T. Koga, M. J. Whitehouse, M. A. Antonelli, J. Farquhar, J. Day, E. H. Hauri, Anomalous sulphur isotopes in plume lavas reveal deep mantle storage of Archaean crust. *Nature* **496**, 490–493 (2013).

116. H. Delavault, C. Chauvel, E. Thomassot, C. W. Devey, B. Dazas, Sulfur and lead isotopic evidence of relic Archean sediments in the Pitcairn mantle plume. *Proc. Natl. Acad. Sci. U.S.A.* **113**, 12952–12956 (2016).

117. E. M. Syracuse, G. A. Abers, Global compilation of variations in slab depth beneath arc volcanoes and implications. *Geochem. Geophys. Geosyst.* **7**, 2005GC001045 (2006).

118. A. Aiuppa, T. P. Fischer, T. Plank, P. Robidoux, R. Di Napoli, Along-arc, inter-arc and arc-to-arc variations in volcanic gas CO_2/ST ratios reveal dual source of carbon in arc volcanism. *Earth Sci. Rev.* **168**, 24–47 (2017).

119. E. Mason, M. Edmonds, A. V. Turchyn, Remobilization of crustal carbon may dominate volcanic arc emissions. *Science* **357**, 290–294 (2017).

120. J. C. Alt, Sulfur isotopic profile through the oceanic crust: Sulfur mobility and seawater-crustal sulfur exchange during hydrothermal alteration. *Geology* **23**, 585–588 (1995).

121. M. J. Mottl, C. G. Wheat, P. Fryer, J. Gharib, J. B. Martin, Chemistry of springs across the Mariana forearc shows progressive devolatilization of the subducting plate. *Geochim. Cosmochim. Acta* **68**, 4915–4933 (2004).

122. M. Mottl, J. Alt, "Data report: Minor and trace element and sulfur isotopic composition of pore waters from Sites 778 through 786," in *Proceedings of the Integrated Ocean Drilling Program: Scientific Results* (Integrated Ocean Drilling Program, 1992), vol. 125.

123. R. C. Newton, C. E. Manning, Solubility of anhydrite, $CaSO_4$, in NaCl-H_2O solutions at high pressures and temperatures: Applications to fluid rock interaction. *J. Petrol.* **46**, 701–716 (2004).

124. D. A. Stolper, J. A. Higgins, L. A. Derry, The role of the solid earth in regulating atmospheric O_2 levels. *Am. J. Sci.* **321**, 1381–1444 (2021).

125. M. M. Hirschmann, The deep Earth oxygen cycle: Mass balance considerations on the origin and evolution of mantle and surface oxidative reservoirs. *Earth Planet. Sci. Lett.* **619**, 118311 (2023).

126. W. W. Fischer, J. Hemp, J. E. Johnson, Evolution of oxygenic photosynthesis. *Annu. Rev. Earth Planet. Sci.* **44**, 647–683 (2016).

127. Z. Peng, C. Wang, X. Tong, L. Zhang, B. Zhang, Element geochemistry and neodymium isotope systematics of the Neoarchean banded iron formations in the Qingyuan greenstone belt, North China Craton. *Ore Geol. Rev.* **102**, 562–584 (2018).

128. D. J. Rasmussen, T. A. Plank, P. J. Wallace, M. E. Newcombe, J. B. Lowenstern, Vapor-bubble growth in olivine-hosted melt inclusions. *Am. Mineral.* **105**, 1898–1919 (2020).

129. D. J. Rasmussen, "The Aleutian Arc through and through: Subduction dynamics and the generation, storage, and eruption of hydrous magmas," thesis, Lamont-Doherty Earth Observatory, Columbia University, 2019.

130. T. A. Plank, Wade, Bulk rock data for Marianas arc tephra, Interdisciplinary Earth Data Alliance [accessed 24 October 2024] (2020).

131. L. B. Cooper, T. Plank, R. J. Arculus, E. H. Hauri, P. S. Hall, S. W. Parman, High-Ca boninites from the active Tonga Arc. *J. Geophys. Res. Solid Earth* **115**, 2009JB006367 (2010).

132. A. Barth, M. Newcombe, T. Plank, H. Gonnermann, S. Hajimirza, G. J. Soto, A. Saballos, E. Hauri, Magma decompression rate correlates with explosivity at basaltic volcanoes—Constraints from water diffusion in olivine. *J. Volcanol. Geotherm. Res.* **387**, 106664 (2019).

133. O. Shorttle, Y. Moussallam, M. E. Hartley, J. Maclennan, M. Edmonds, B. J. Murton, Fe-XANES analyses of Reykjanes Ridge basalts: Implications for oceanic crust's role in the solid Earth oxygen cycle. *Earth Planet. Sci. Lett.* **427**, 272–285 (2015).

134. A. W. Hofmann, Chemical differentiation of the Earth: The relationship between mantle, continental crust, and oceanic crust. *Earth Planet. Sci. Lett.* **90**, 297–314 (1988).

135. H. Palme, H. S. C. O'Neill, Cosmochemical estimates of mantle composition. *Treat. Geochem.* **2**, 1–38 (2003).

136. A. Paytan, M. Kastner, D. Campbell, M. H. Thiemens, Sulfur isotopic composition of Cenozoic seawater sulfate. *Science* **282**, 1459–1462 (1998).

137. K. A. Kelley, T. Plank, T. L. Grove, E. M. Stolper, S. Newman, E. Hauri, Mantle melting as a function of water content beneath back-arc basins. *J. Geophys. Res. Solid Earth* **111**, 2005JB003732 (2006).

138. C. E. Bucholz, G. A. Gaetani, M. D. Behn, N. Shimizu, Post-entrapment modification of volatiles and oxygen fugacity in olivine-hosted melt inclusions. *Earth Planet. Sci. Lett.* **374**, 145–155 (2013).

139. J. D. Blundy, T. J. Falloon, B. J. Wood, J. A. Dalton, Sodium partitioning between clinopyroxene and silicate melts. *J. Geophys. Res. Solid Earth* **100**, 15501–15515 (1995).

140. G. Mallmann, H. S. C. O'Neill, Calibration of an empirical thermometer and oxybarometer based on the partitioning of Sc, Y and V between olivine and silicate melt. *J. Petrol.* **54**, 933–949 (2013).

141. Z. Taracsák, D. Neave, P. Beaudry, J. Gunnarsson-Robin, R. Burgess, M. Edmonds, S. Halldórsson, M.-A. Longpré, S. Ono, E. Ranta, A. Stefánsson, A. Turchyn, EIMF, M. Hartley, Instrumental mass fractionation during sulfur isotope analysis by secondary ion mass spectrometry in natural and synthetic glasses. *Chem. Geol.* **578**, 120318 (2021).

142. T. Plank, Constraints from thorium/lanthanum on sediment recycling at subduction zones and the evolution of the continents. *J. Petrol.* **46**, 921–944 (2005).

143. M. Reagan, E. Duarte, G. J. Soto, E. Fernández, The eruptive history of Turrialba volcano, Costa Rica, and potential hazards from future eruptions. *Geol. Soc. Am. Spec. Pap.* **412**, 235 (2006).

144. A. S. Lloyd, T. Plank, P. Ruprecht, E. H. Hauri, W. Rose, Volatile loss from melt inclusions in pyroclasts of differing sizes. *Contrib. Mineral. Petrol.* **165**, 129–153 (2013).

145. A. Di Piazza, A. Rizzo, F. Barberi, M. Carapezza, G. De Astis, C. Romano, F. Sortino, Geochemistry of the mantle source and magma feeding system beneath Turrialba volcano, Costa Rica. *Lithos* **232**, 319–335 (2015).

146. J. M. de Moor, A. Aiuppa, G. Avard, H. Wehrmann, N. Dunbar, C. Muller, G. Tamburello, G. Giudice, M. Liuzzo, R. Moretti, V. Conde, B. Galle, Turmoil at Turrialba Volcano (Costa Rica): Degassing and eruptive processes inferred from high-frequency gas monitoring. *J. Geophys. Res. Solid Earth* **121**, 5761–5775 (2016).

147. M. Carr, M. Feigenson, L. Bolge, J. Walker, E. Gazel, RU_CAGeochem v.4, a database and sample repository for Central American volcanic rocks at Rutgers University. *Geosci. Data J.* **1**, 43–48 (2015).

148. T. H. Dixon, R. Batiza, Petrology and chemistry of recent lavas in the northern Marianas: Implications for the origin of island arc basalts. *Contrib. Mineral. Petrol.* **70**, 167–181 (1979).

149. R. J. Stern, On the origin of andesite in the northern Mariana island arc: Implications from Agrigan. *Contrib. Mineral. Petrol.* **68**, 207–219 (1979).

150. A. Meijer, M. Reagan, Petrology and geochemistry of the island of Sarigan in the Mariana Arc; calc-alkaline volcanism in an oceanic setting. *Contrib. Mineral. Petrol.* **77**, 337–354 (1981).

151. M. J. Hole, A. D. Saunders, G. F. Marriner, J. Tarney, Subduction of pelagic sediments: Implications for the origin of Ce-anomalous basalts from the Mariana Islands. *J. Geol. Soc. London* **141**, 453–472 (1984).

152. J. Woodhead, The origin of geochemical variations in Mariana lavas: A general model for petrogenesis in intra-oceanic island arcs? *J. Petrol.* **29**, 805–830 (1988).

153. J. Woodhead, Geochemistry of the Mariana arc (western Pacific): Source composition and processes. *Chem. Geol.* **76**, 1–24 (1989).

154. T. Elliott, T. Plank, A. Zindler, W. White, B. Bourdon, Element transport from slab to volcanic front at the Mariana arc. *J. Geophys. Res. Solid Earth* **102**, 14991–15019 (1997).
